# Supplementary figures and images for: Cyproterone Acetate Mediates IRE1α Signaling Pathway to Alleviate Pyroptosis of Ovarian Granulosa Cells Induced by Hyperandrogen
Source: Biology (Basel). 2022 Dec 4;11(12):1761. doi: 10.3390/biology11121761 (PMC9775519; doi:10.3390/biology11121761)

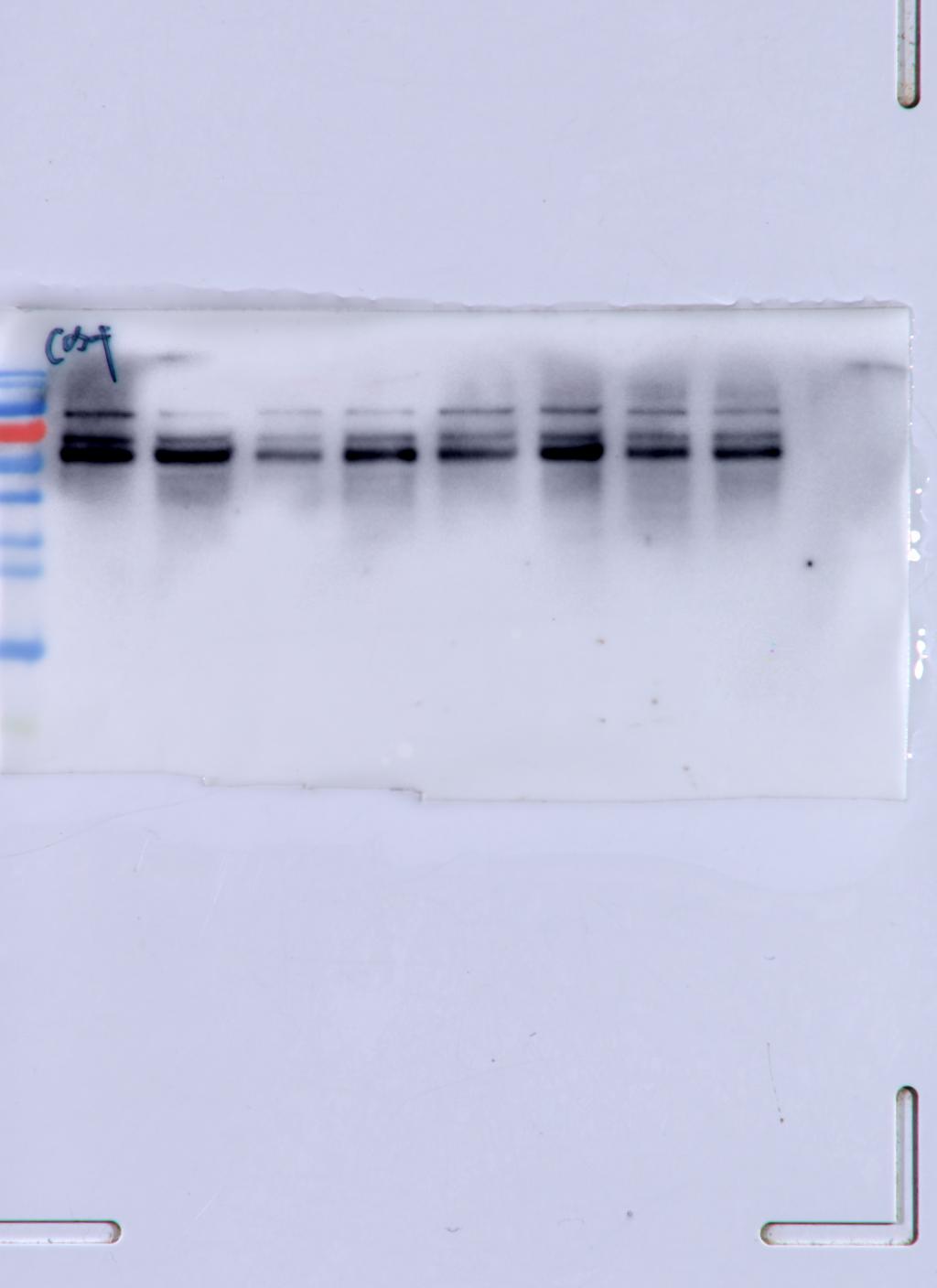

Supplement: Supplementary file 1 [file biology-11-01761-s001.zip › File S1/caspase-1.jpg]

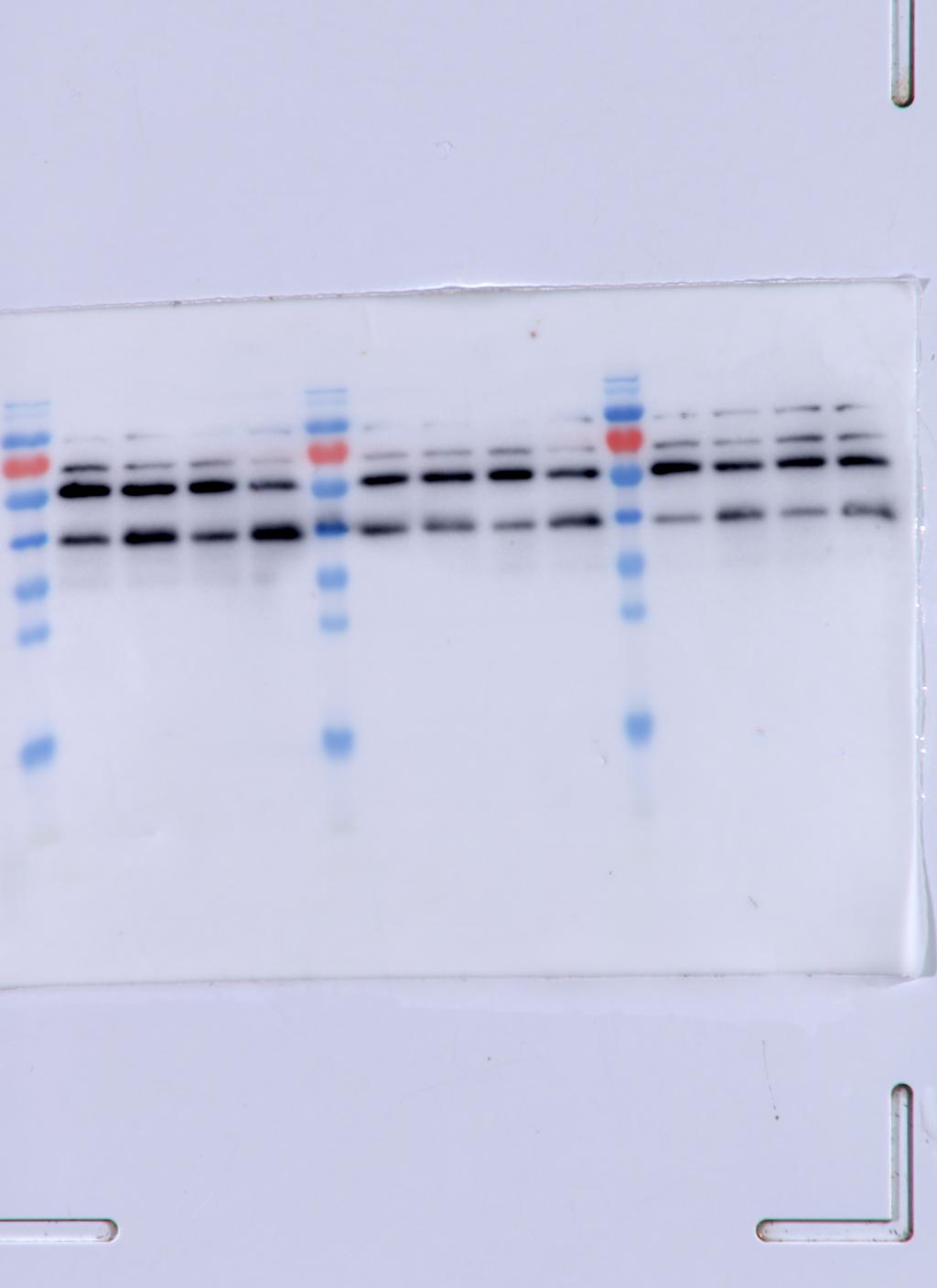

Supplement: Supplementary file 1 [file biology-11-01761-s001.zip › File S1/cleave-caspase-1.jpg]

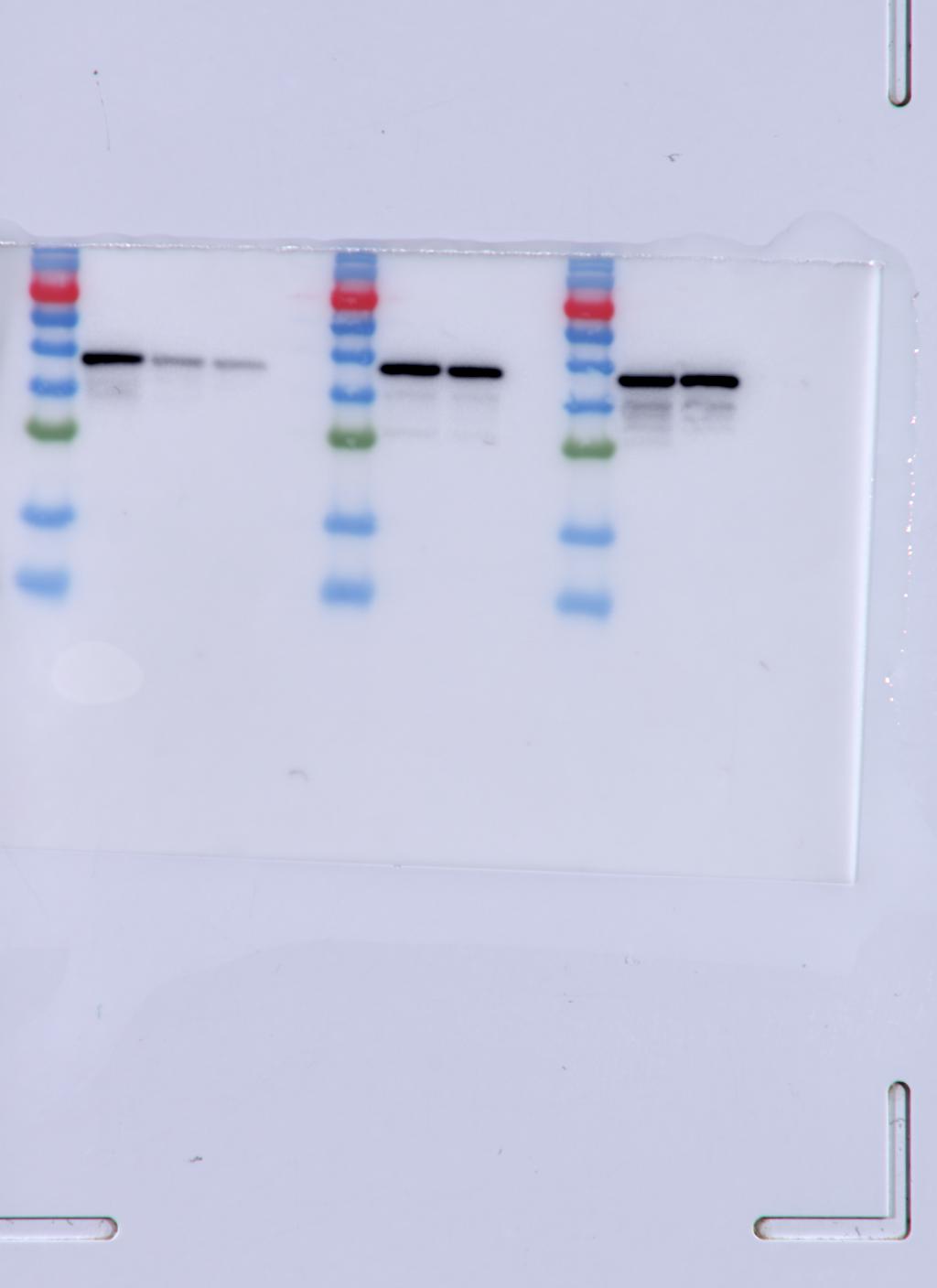

Supplement: Supplementary file 1 [file biology-11-01761-s001.zip › File S1/gapdh1.jpg]

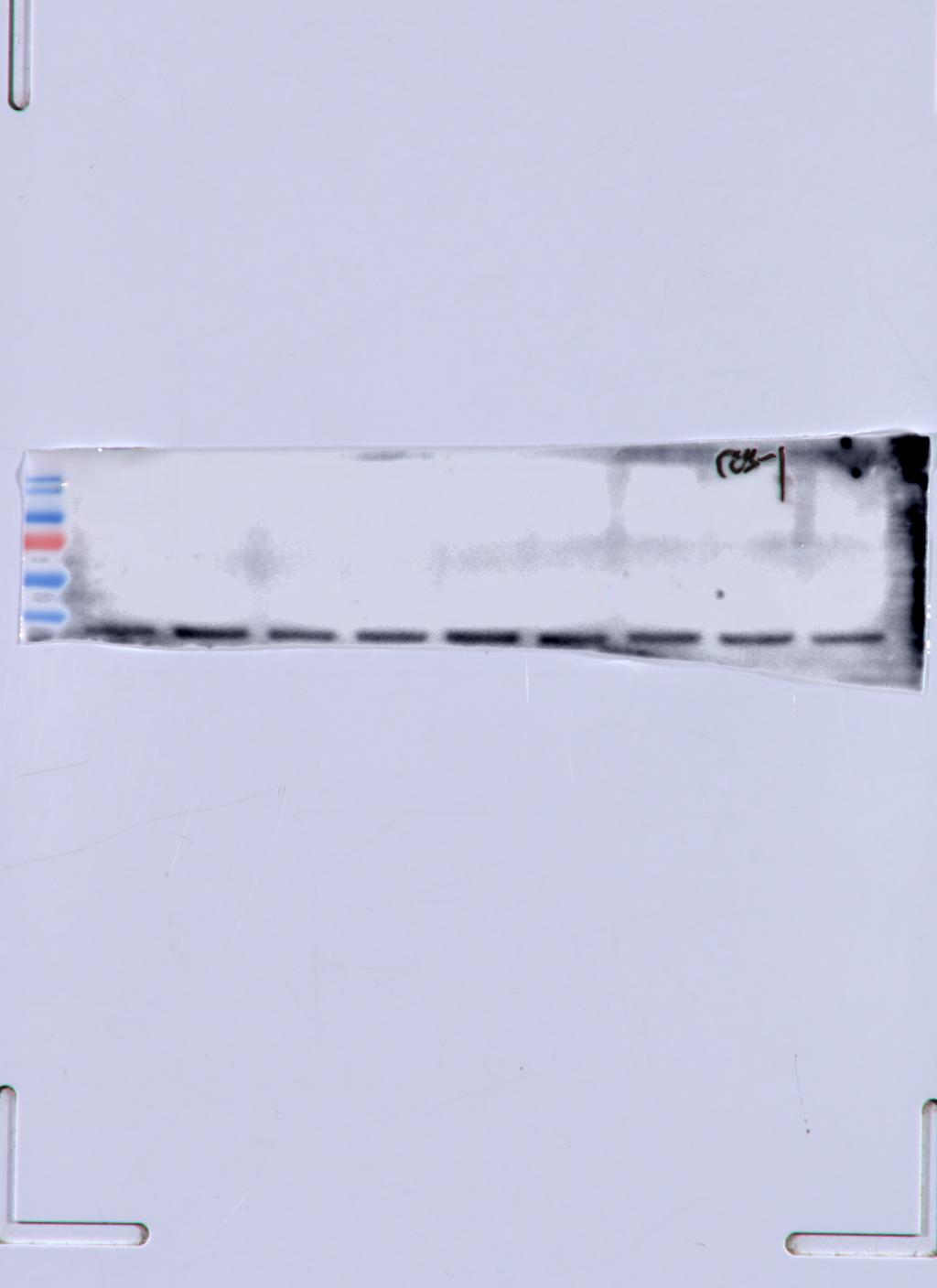

Supplement: Supplementary file 1 [file biology-11-01761-s001.zip › File S1/gapdh2.jpg]

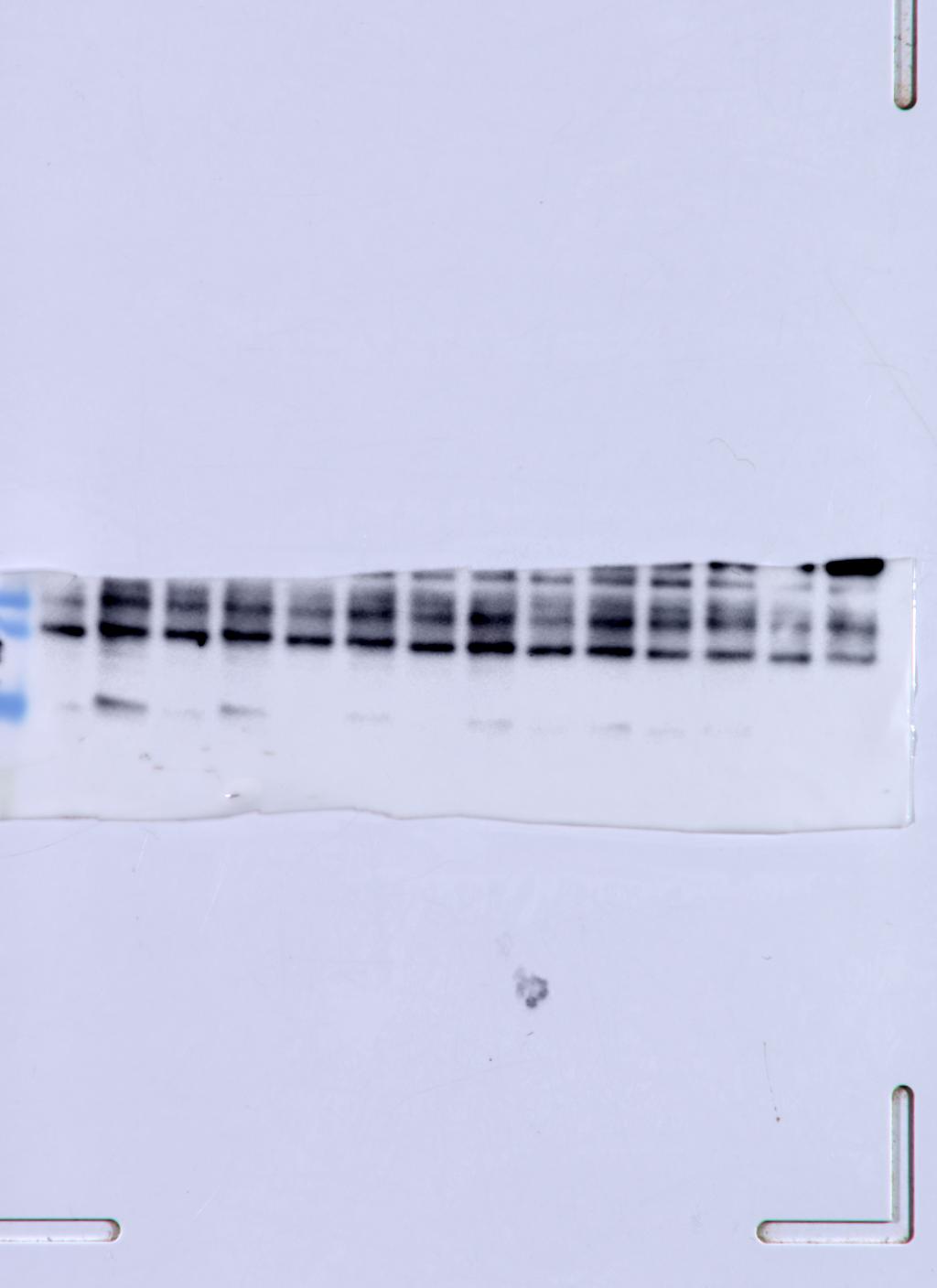

Supplement: Supplementary file 1 [file biology-11-01761-s001.zip › File S1/GD-N.jpg]

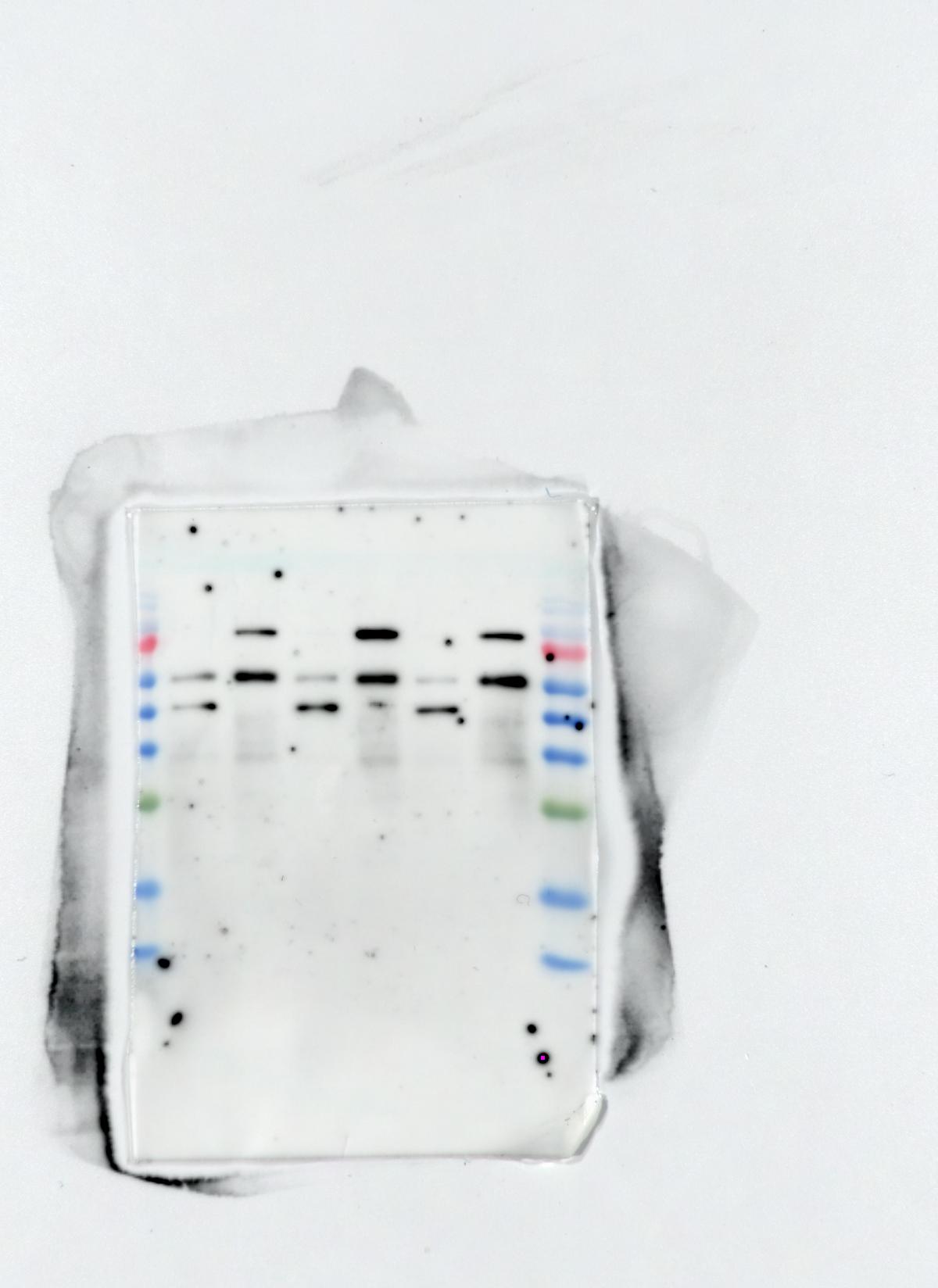

Supplement: Supplementary file 1 [file biology-11-01761-s001.zip › File S1/gd.jpg]

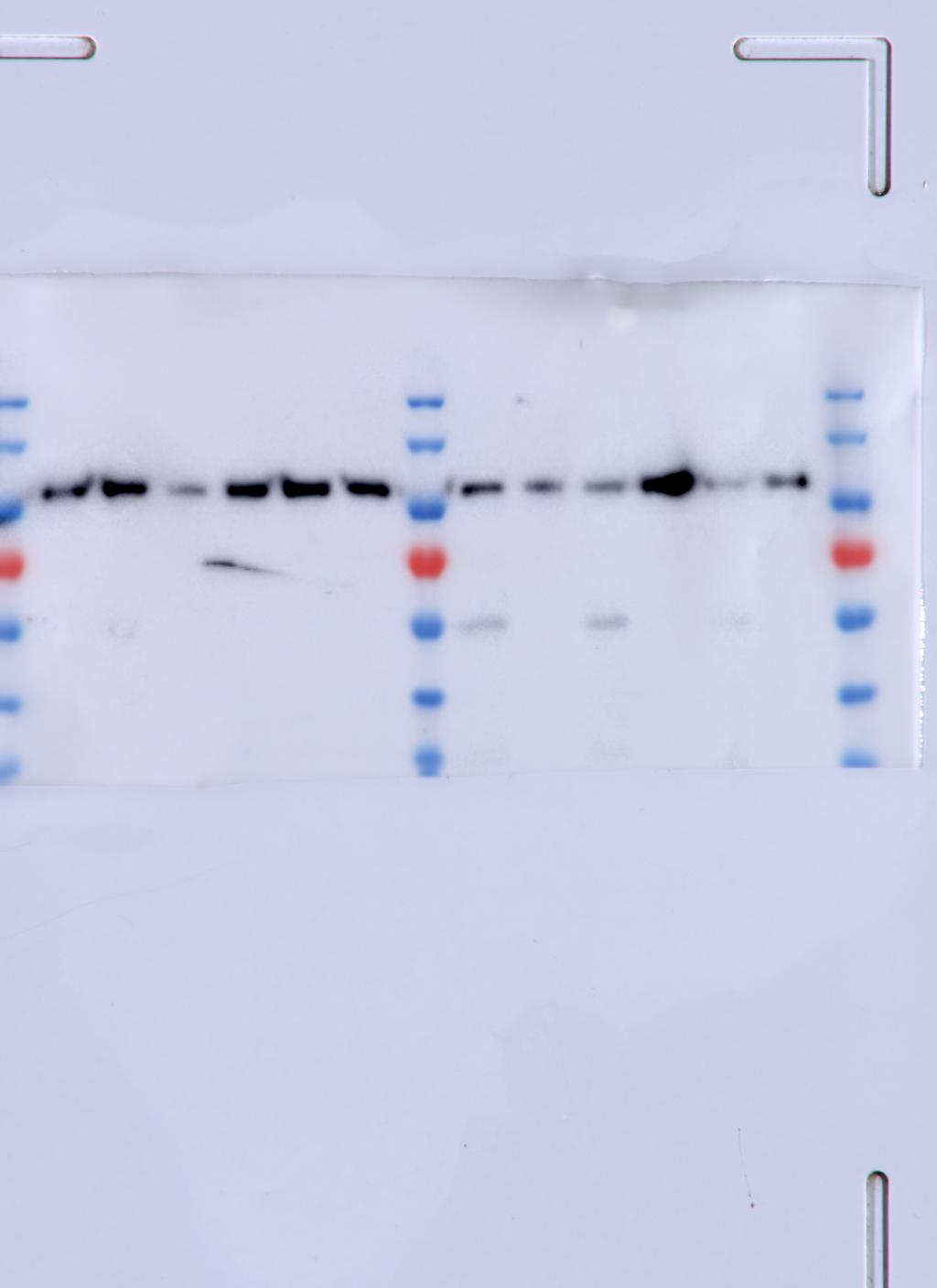

Supplement: Supplementary file 1 [file biology-11-01761-s001.zip › File S1/nlrp3.jpg]

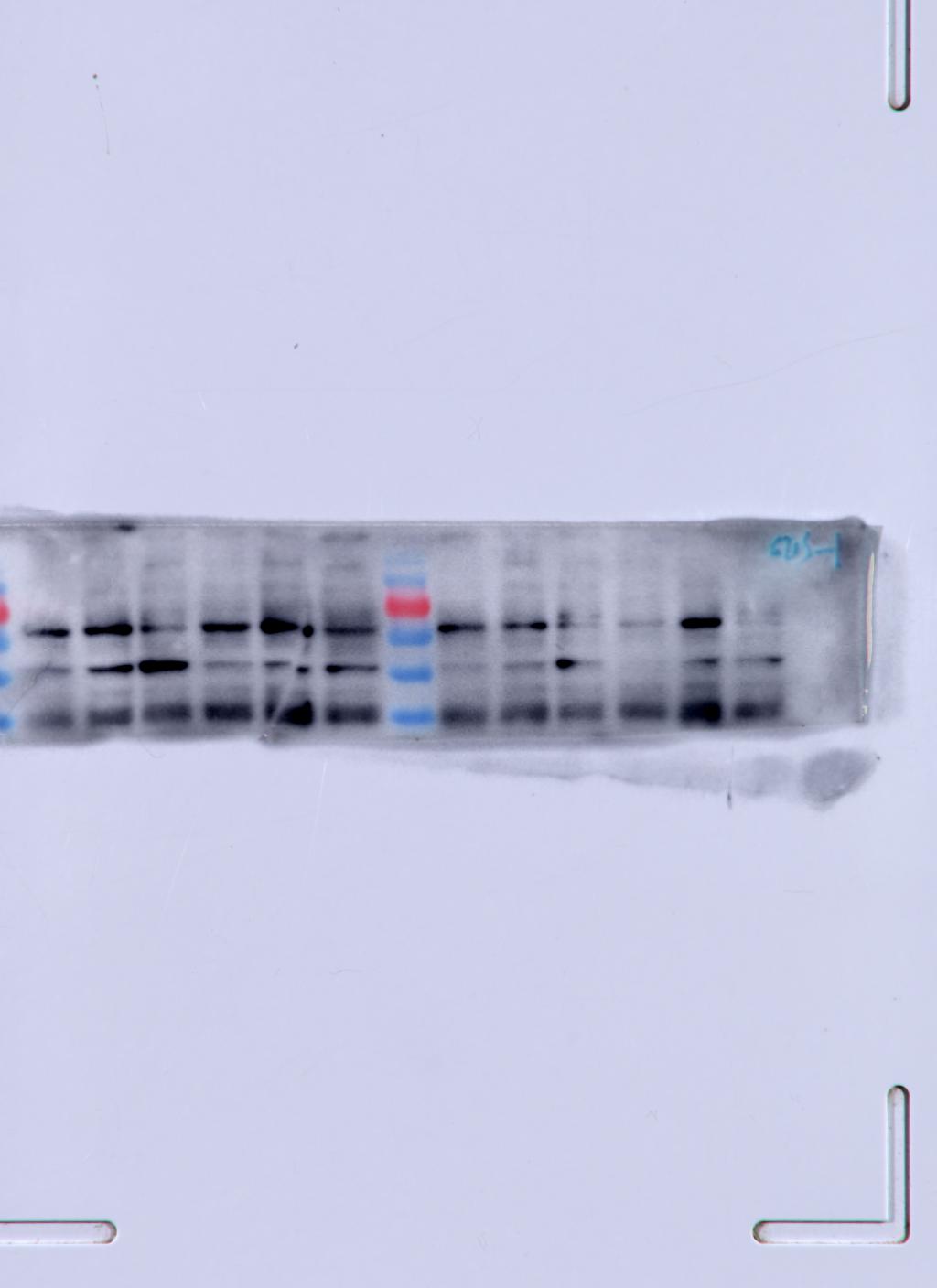

Supplement: Supplementary file 1 [file biology-11-01761-s001.zip › File S2/cCaspase-1andCaspase-1/cas-1íóccas-1.jpg]

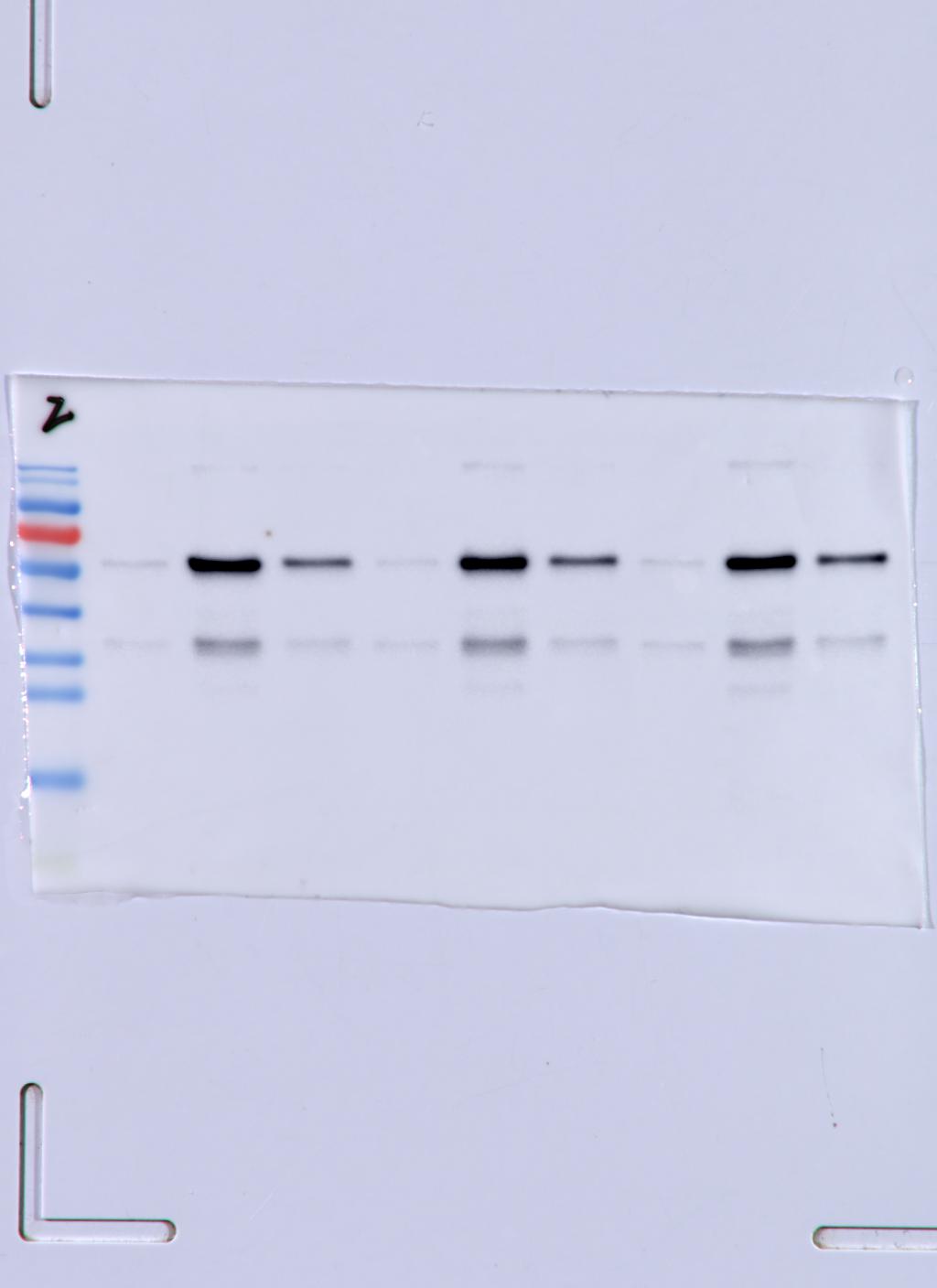

Supplement: Supplementary file 1 [file biology-11-01761-s001.zip › File S2/cCaspase-1andCaspase-1/cas-1íóccas-1ok.jpg]

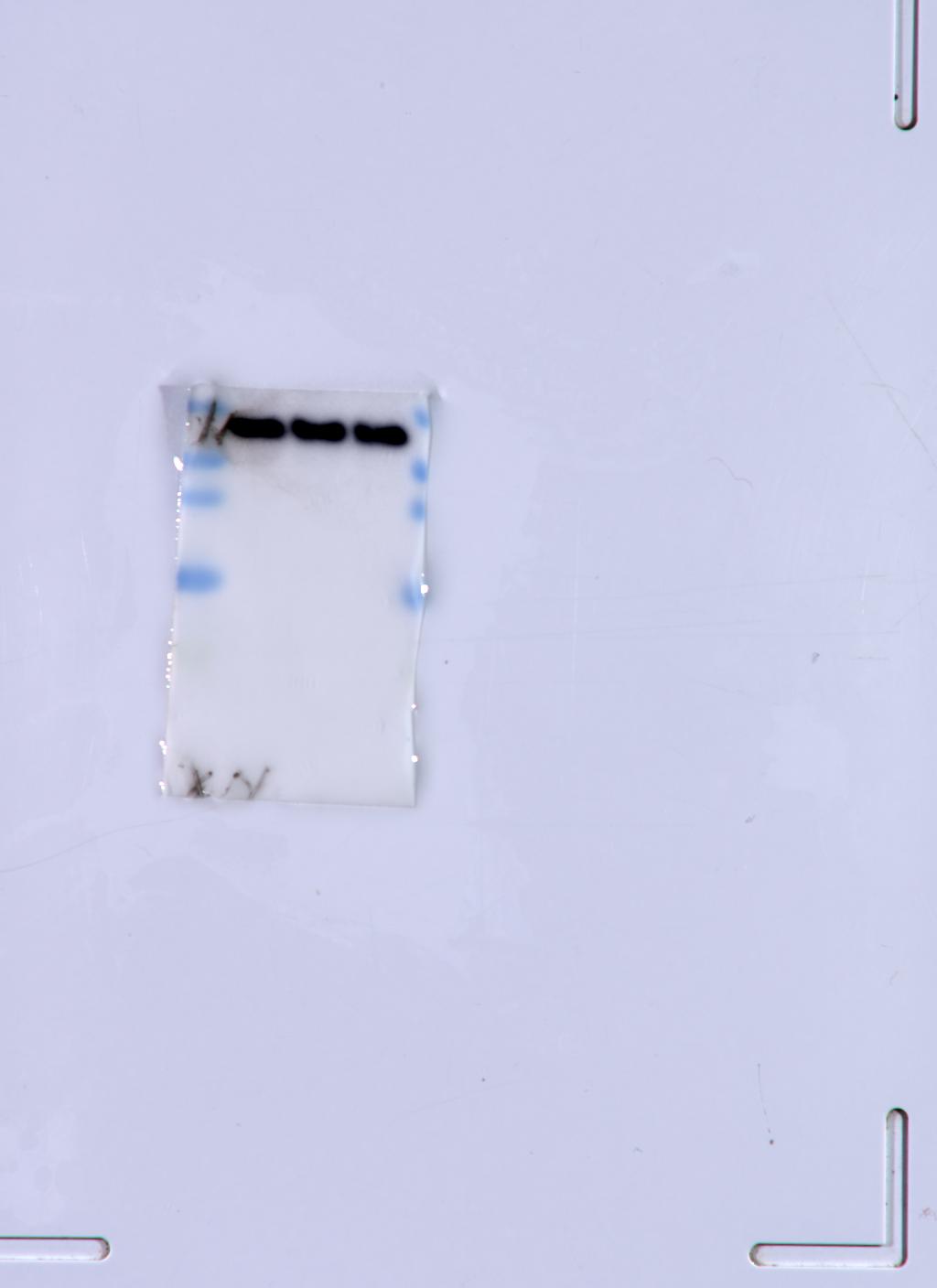

Supplement: Supplementary file 1 [file biology-11-01761-s001.zip › File S2/GAPDH/ngap2 2021.08.18_20.44.05_Ch+Marker.jpg]

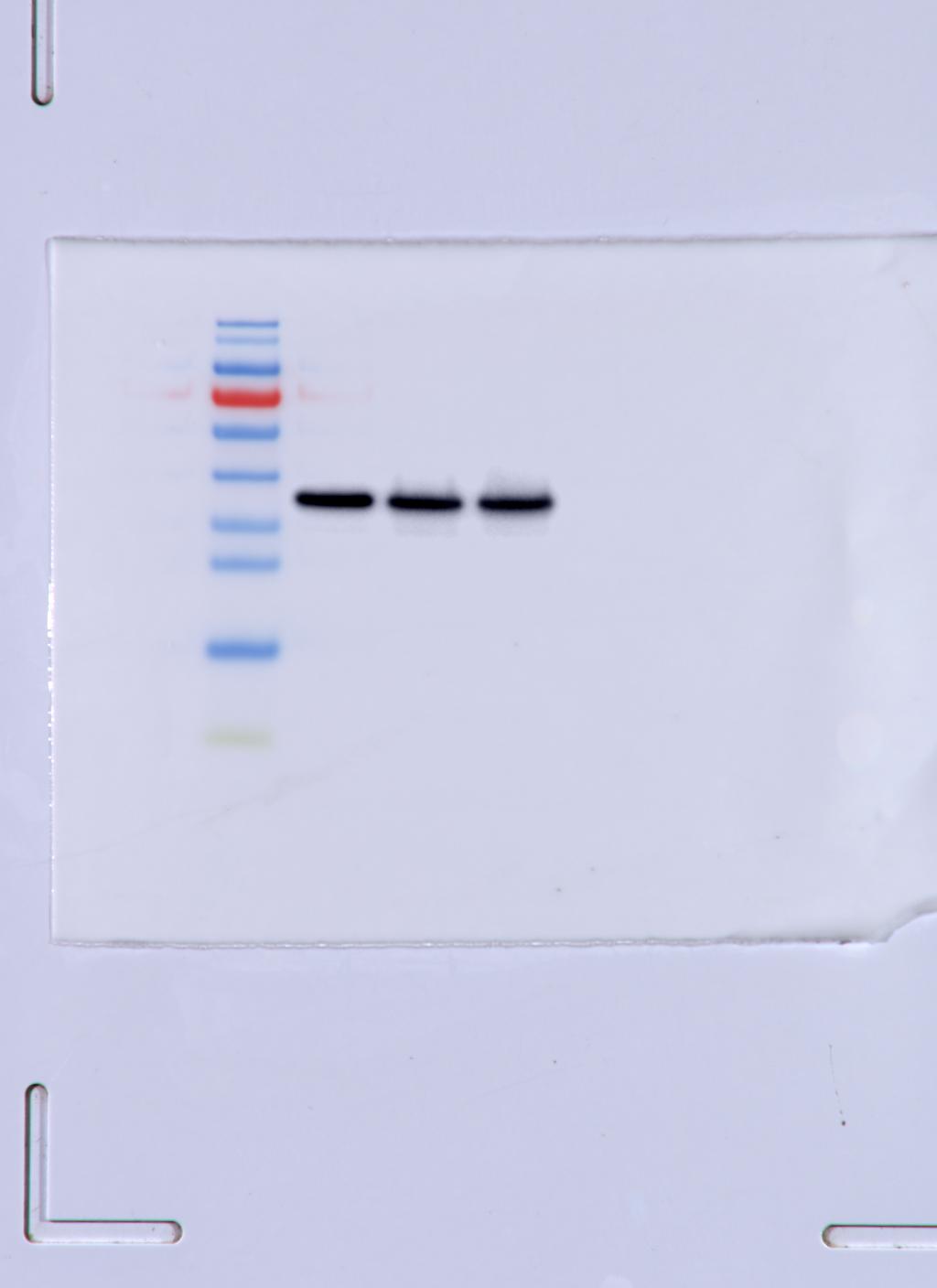

Supplement: Supplementary file 1 [file biology-11-01761-s001.zip › File S2/GAPDH/zy gapdh 10.11 2021.10.11_20.24.55_Ch+Marker.jpg]

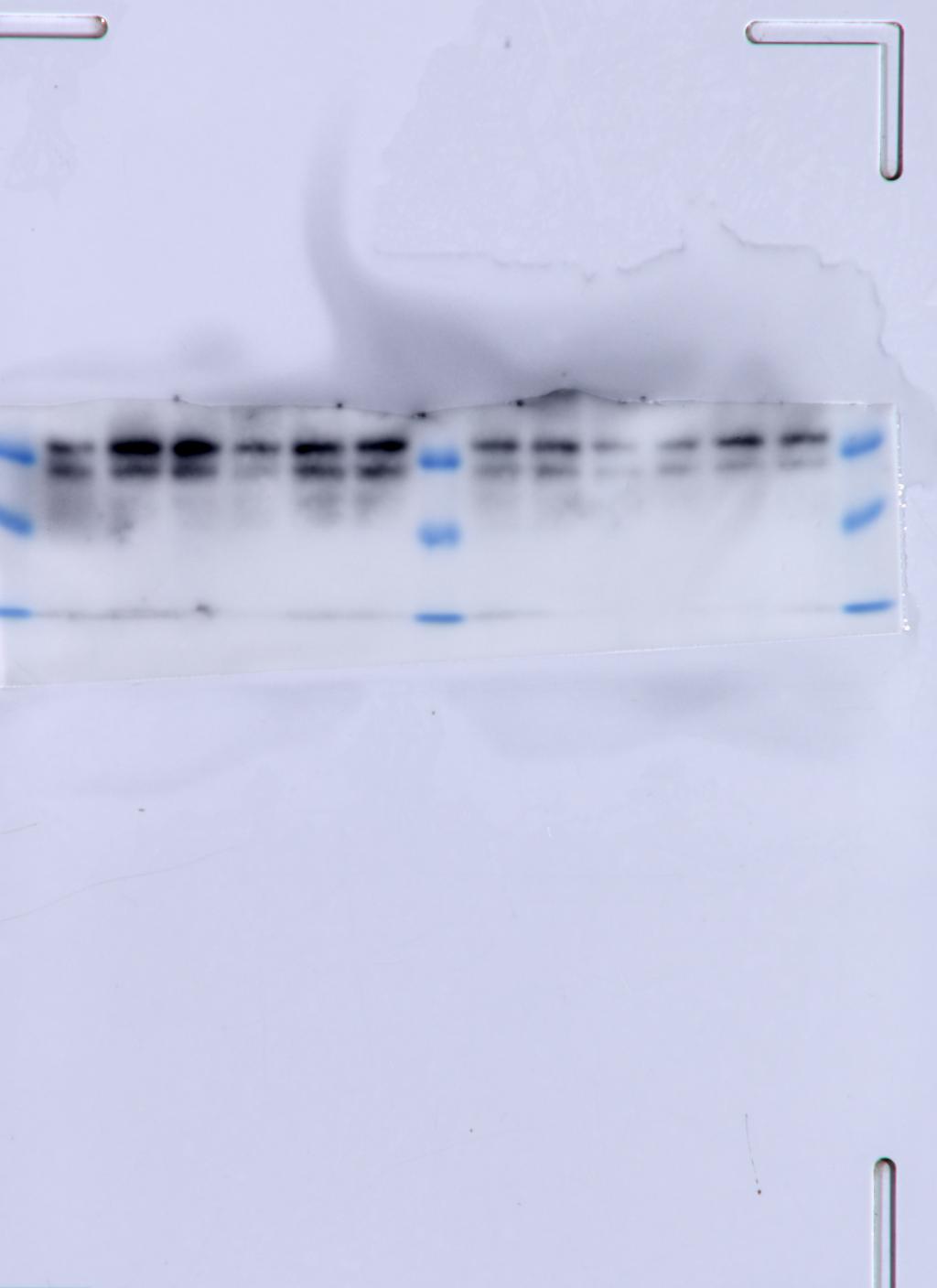

Supplement: Supplementary file 1 [file biology-11-01761-s001.zip › File S2/GD-N/cgd1.jpg]

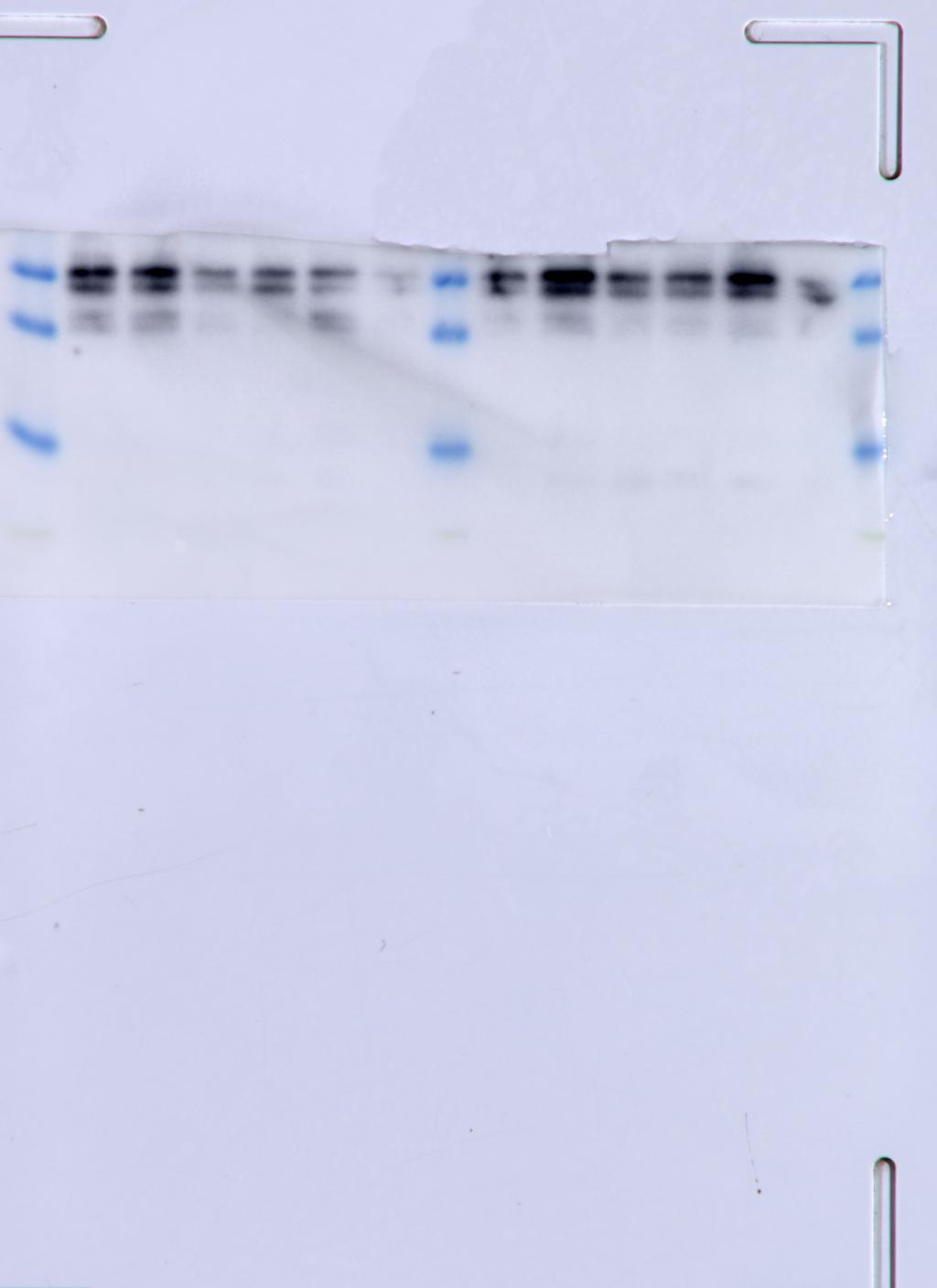

Supplement: Supplementary file 1 [file biology-11-01761-s001.zip › File S2/GD-N/cgd2.jpg]

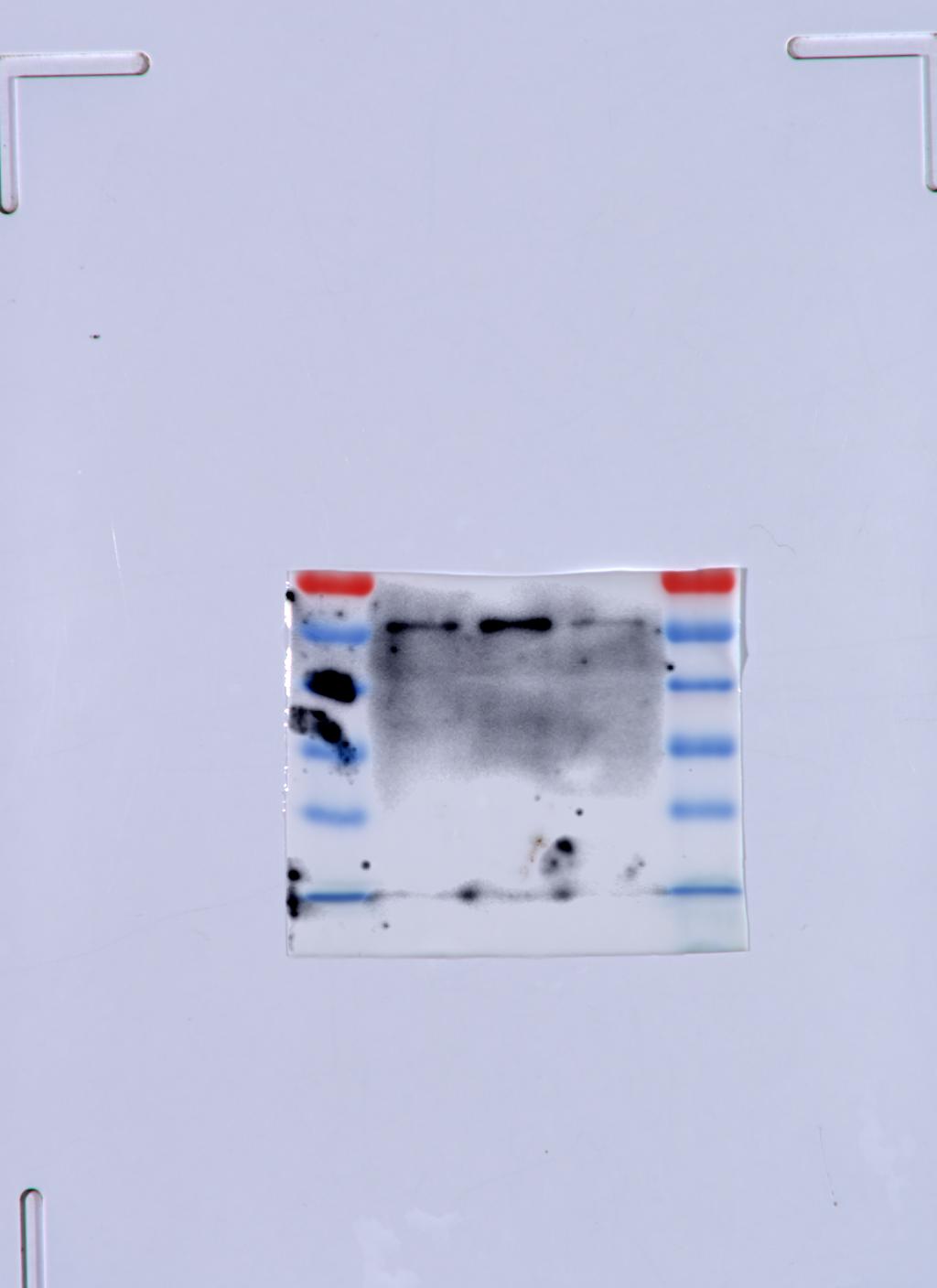

Supplement: Supplementary file 1 [file biology-11-01761-s001.zip › File S2/GD/gd1╕÷ 2021.05.03_17.08.01_Ch+Marker.jpg]

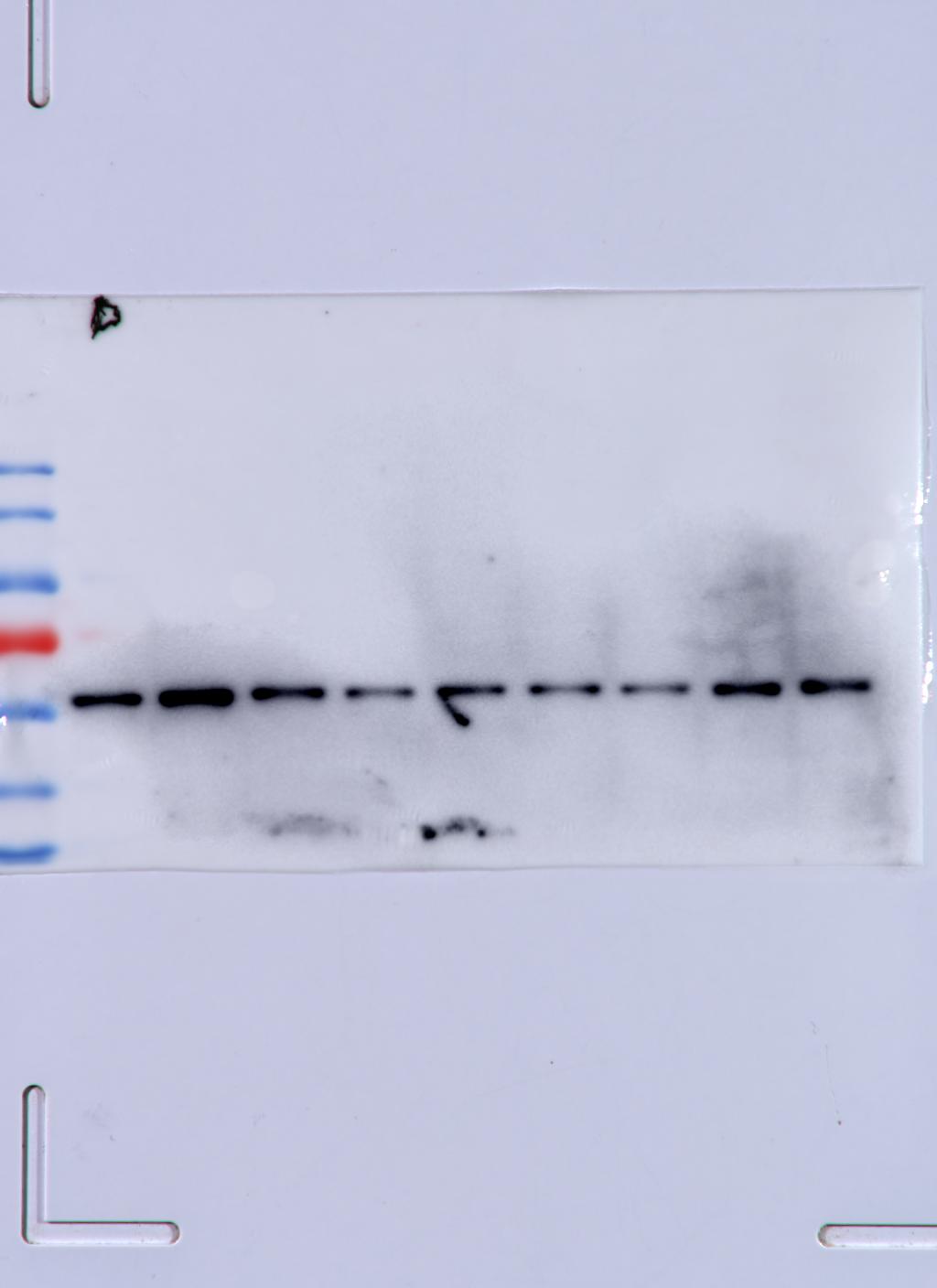

Supplement: Supplementary file 1 [file biology-11-01761-s001.zip › File S2/GD/gd3╕÷ 2021.06.02_15.10.49_Ch+Marker.jpg]

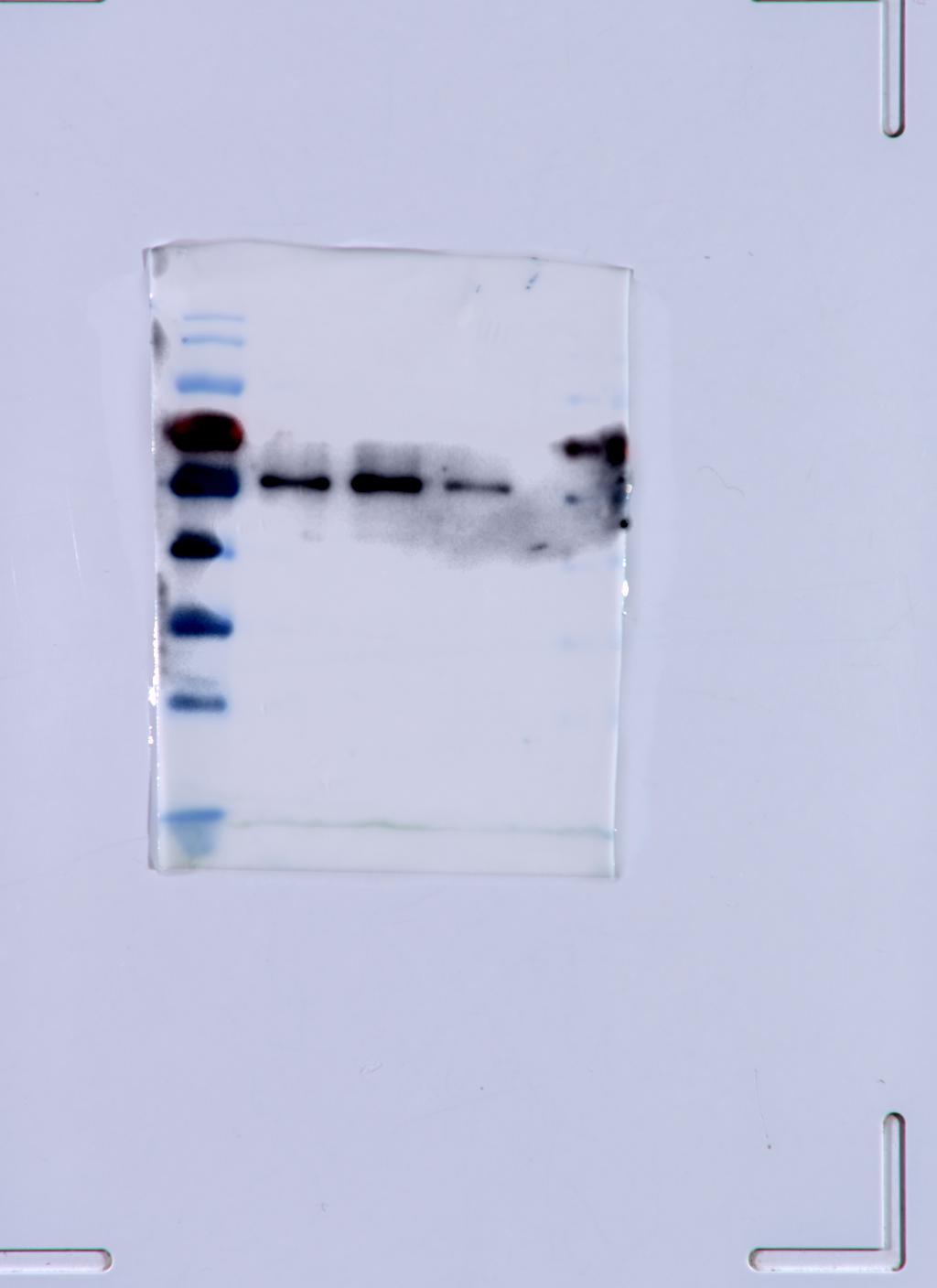

Supplement: Supplementary file 1 [file biology-11-01761-s001.zip › File S2/GD/gd4 2021.05.06_17.11.11_Ch+Marker.jpg]

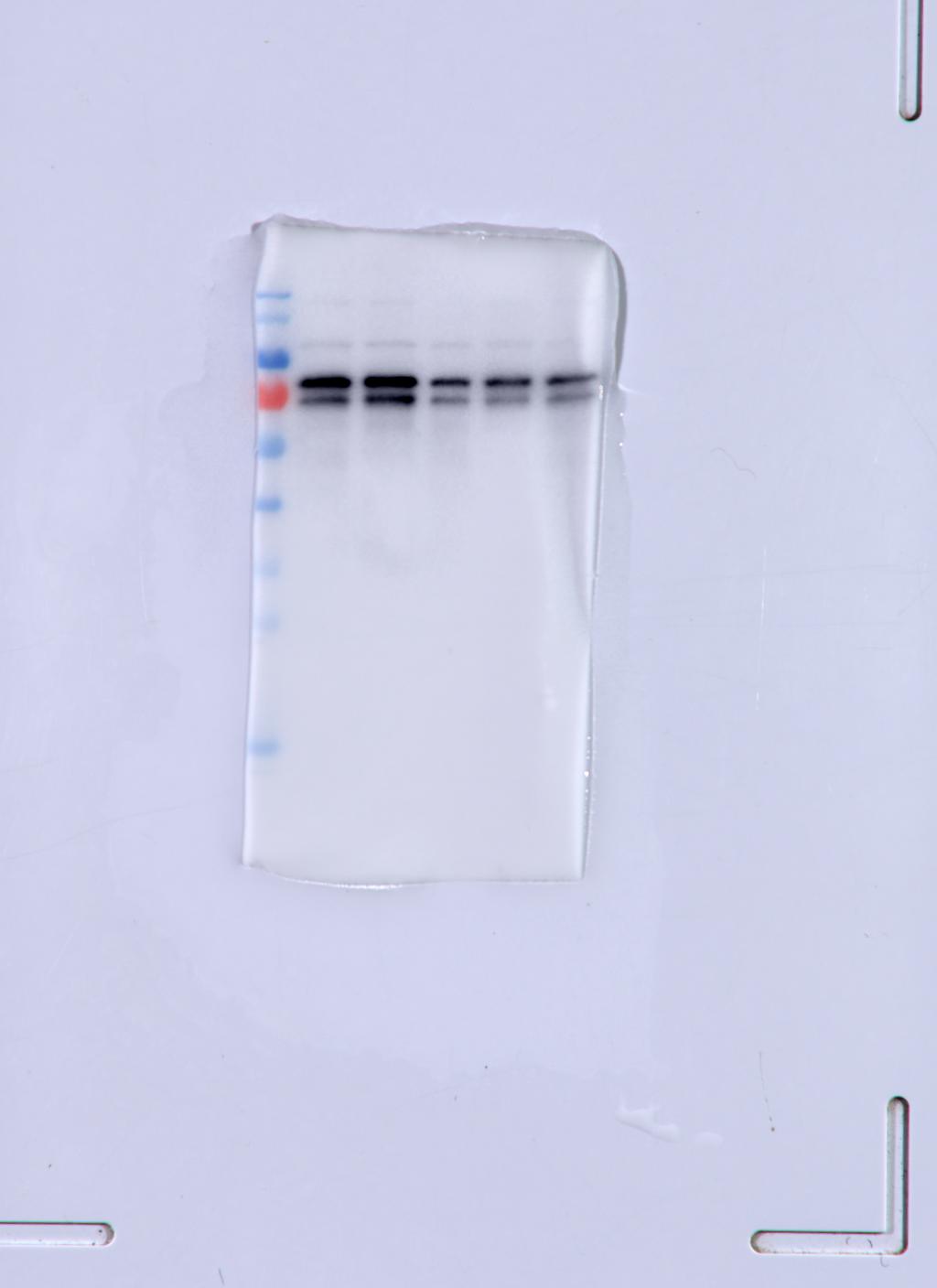

Supplement: Supplementary file 1 [file biology-11-01761-s001.zip › File S2/GRP78/781.jpg]

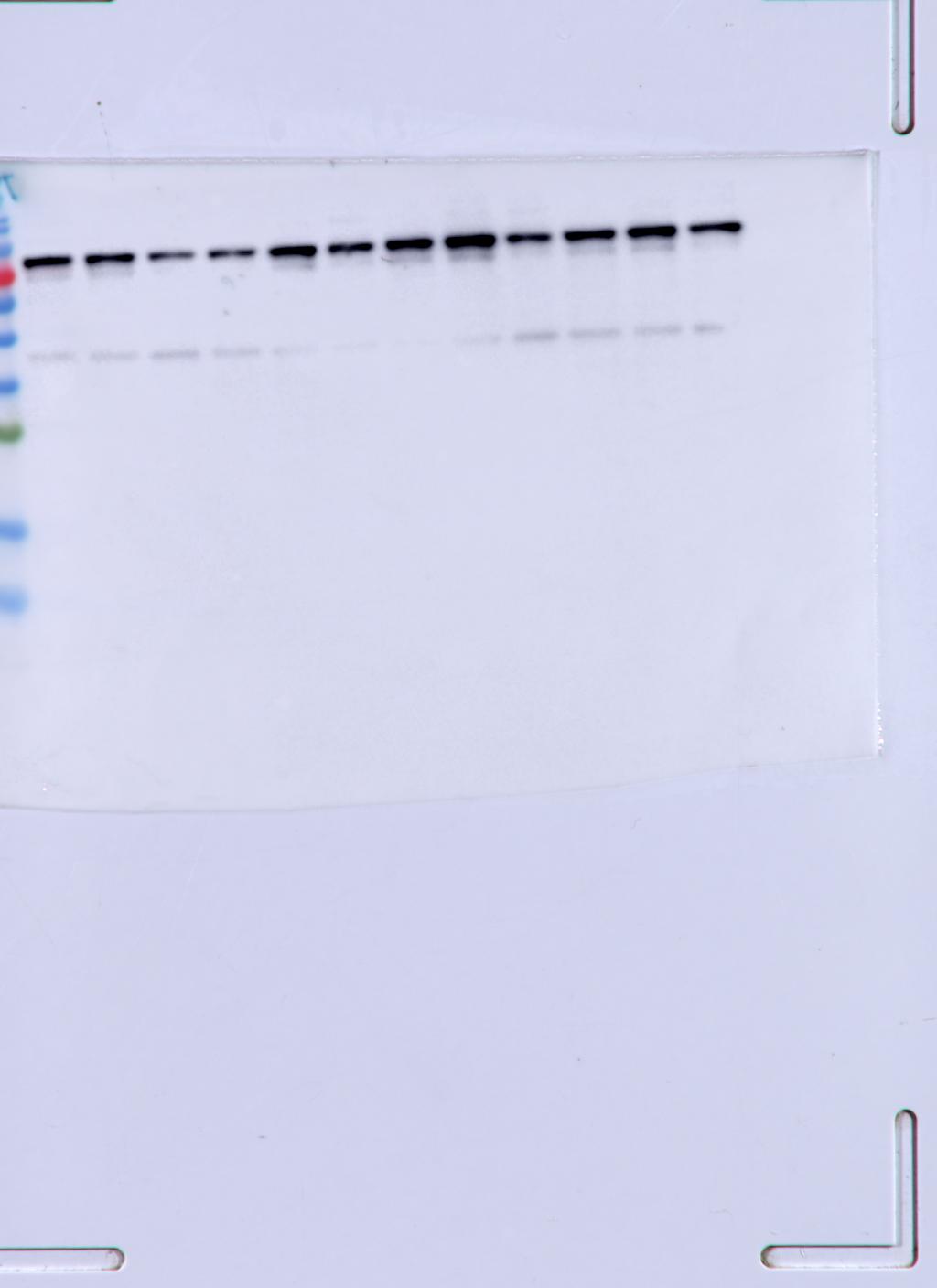

Supplement: Supplementary file 1 [file biology-11-01761-s001.zip › File S2/GRP78/grp-78 2.jpg]

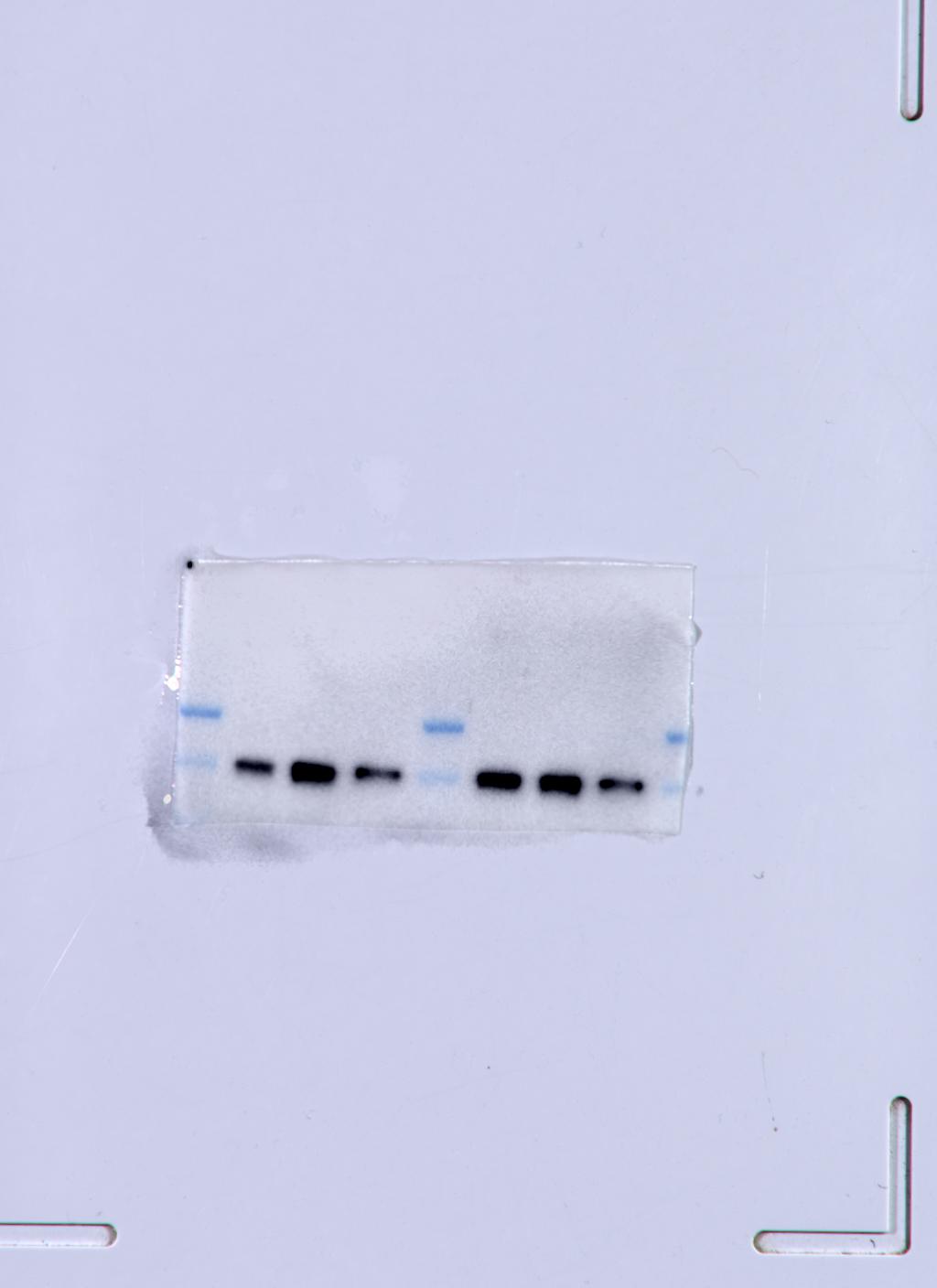

Supplement: Supplementary file 1 [file biology-11-01761-s001.zip › File S2/IRE1/ire111 2022.04.03_17.00.57_Ch+Marker.jpg]

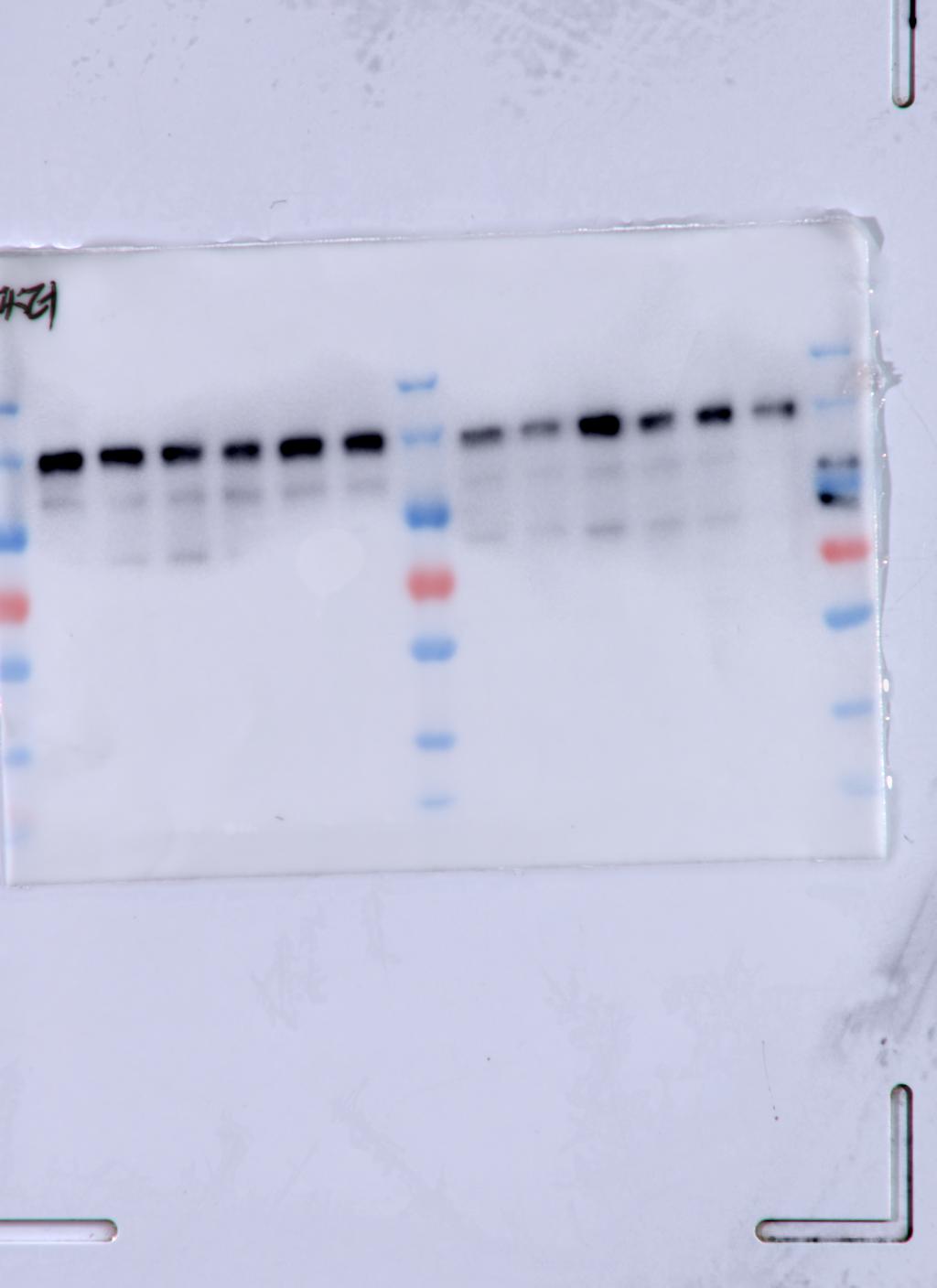

Supplement: Supplementary file 1 [file biology-11-01761-s001.zip › File S2/IRE1/ire111 ok2022.03.27_19.36.16_Ch+Marker.jpg]

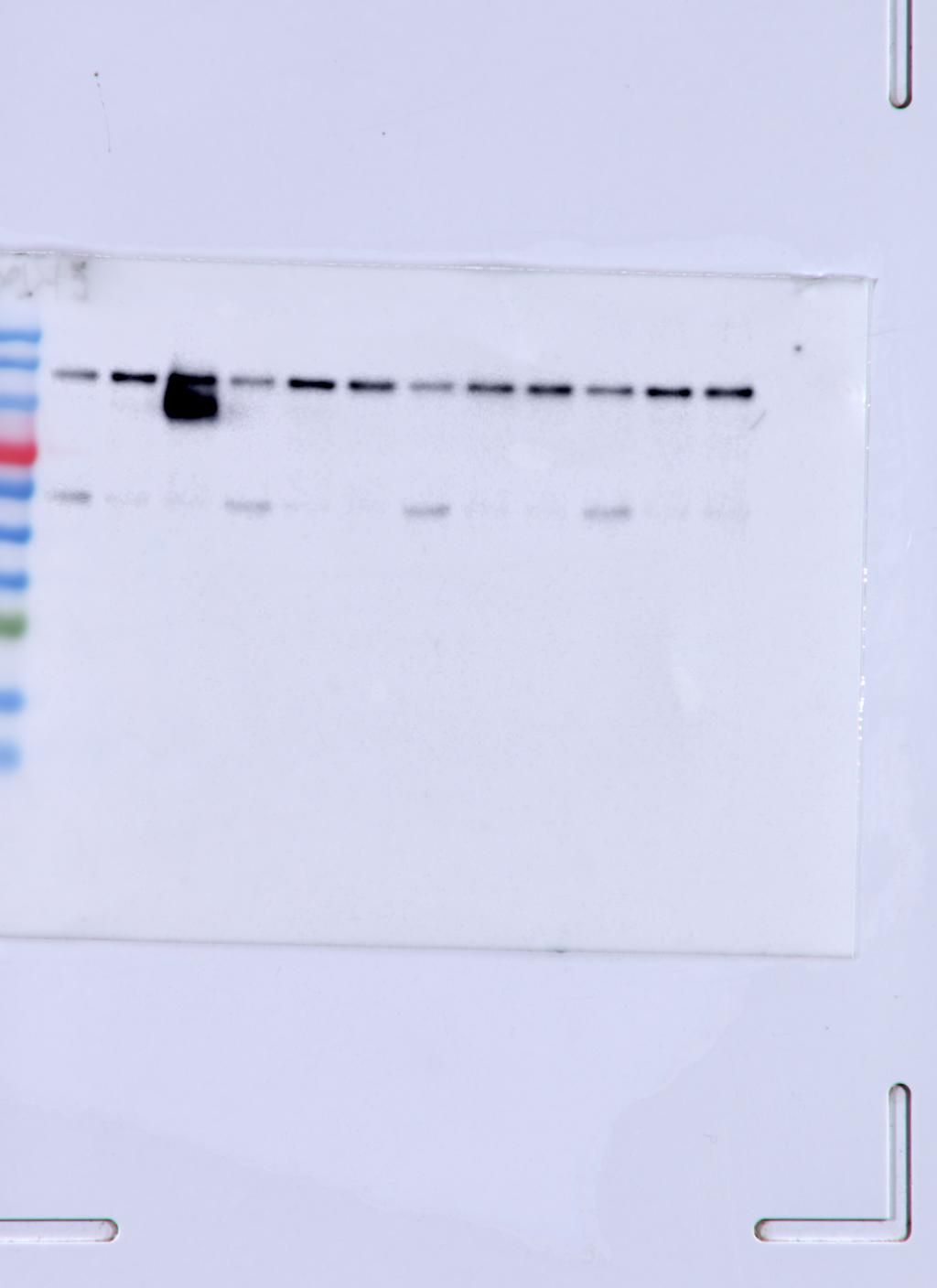

Supplement: Supplementary file 1 [file biology-11-01761-s001.zip › File S2/IRE1/ire2 2021.12.26_16.16.16_Ch+Marker.jpg]

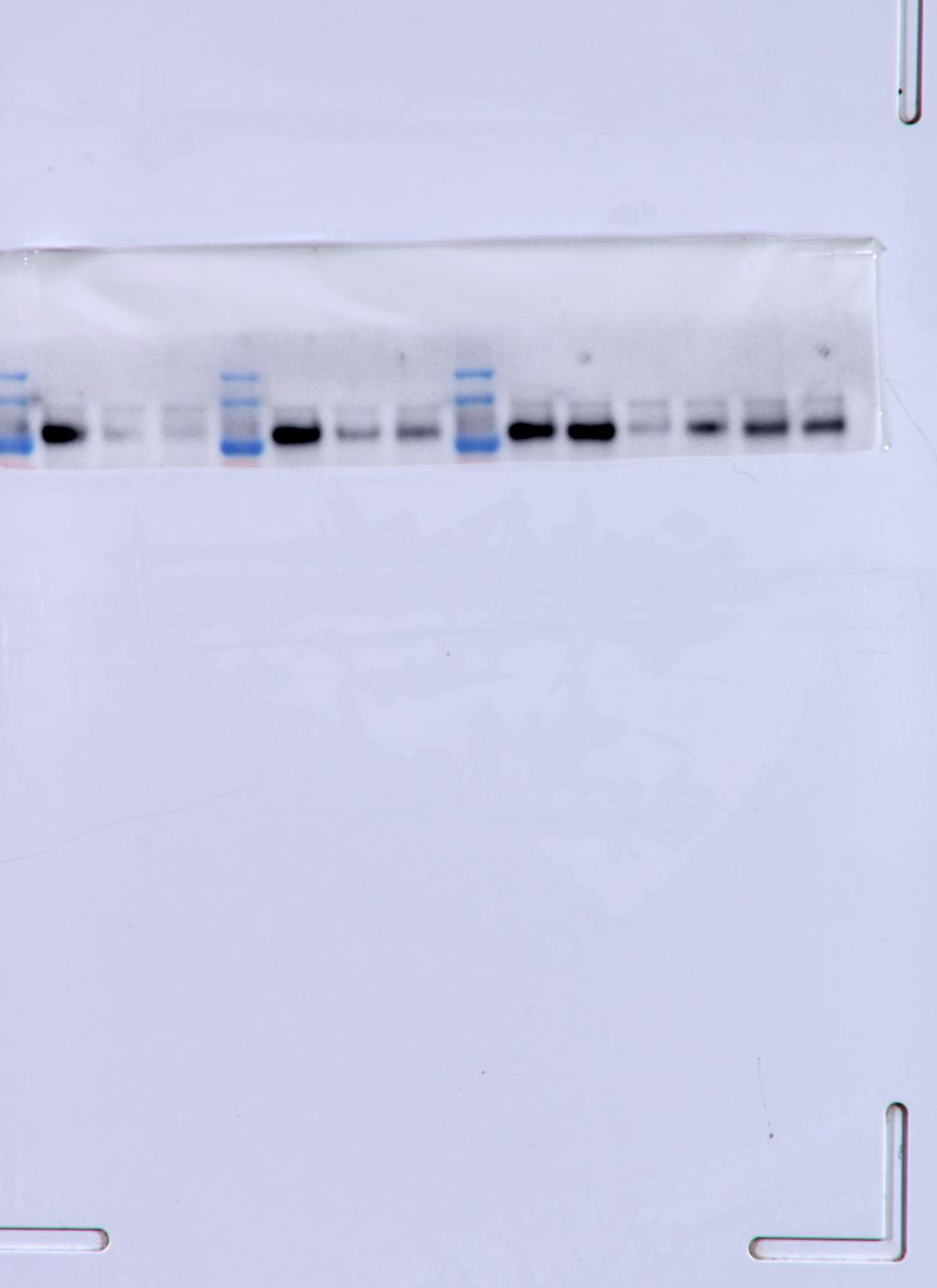

Supplement: Supplementary file 1 [file biology-11-01761-s001.zip › File S2/nlrp3/nlrp3-1.jpg]

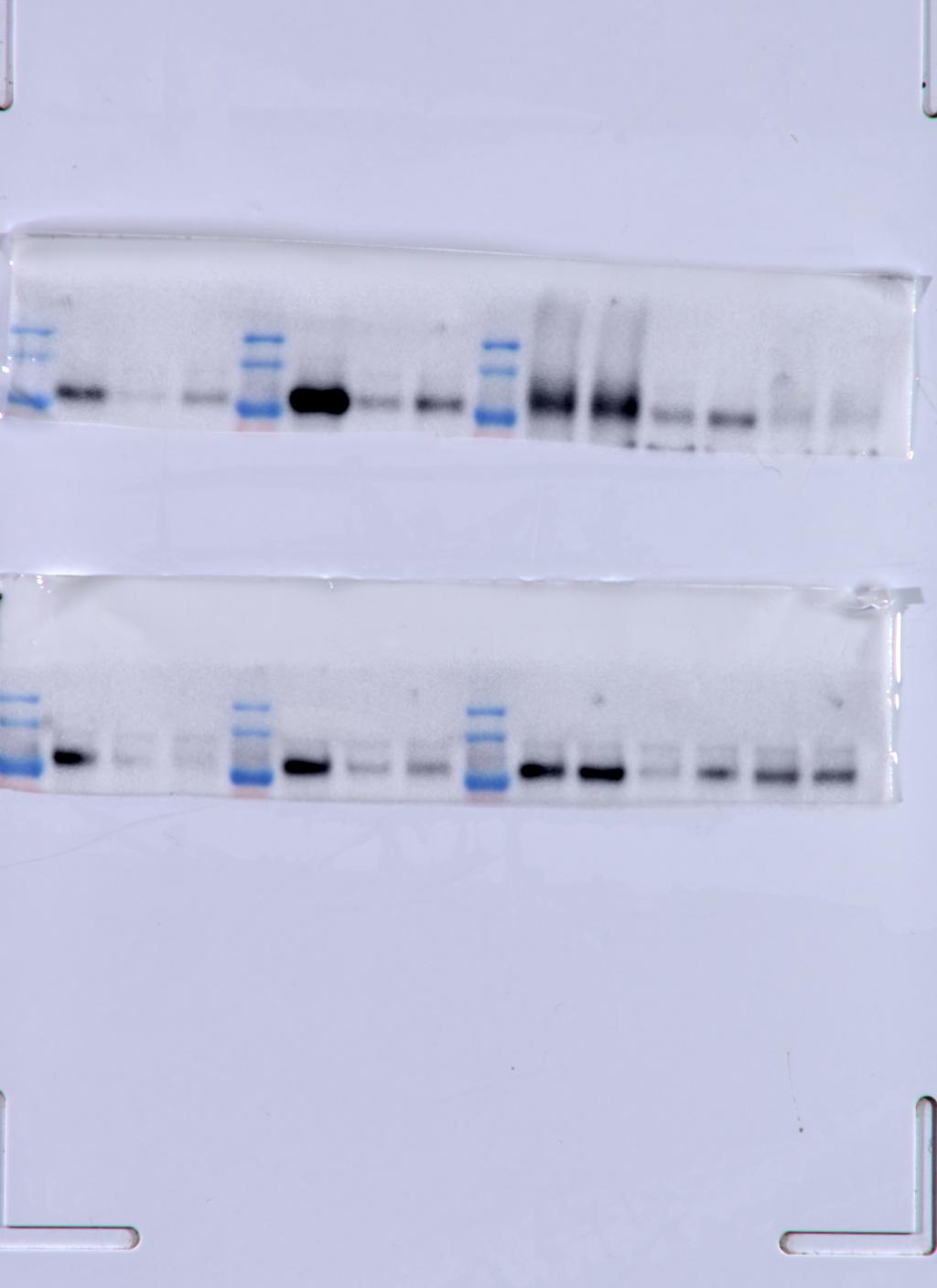

Supplement: Supplementary file 1 [file biology-11-01761-s001.zip › File S2/nlrp3/nlrp3-2.jpg]

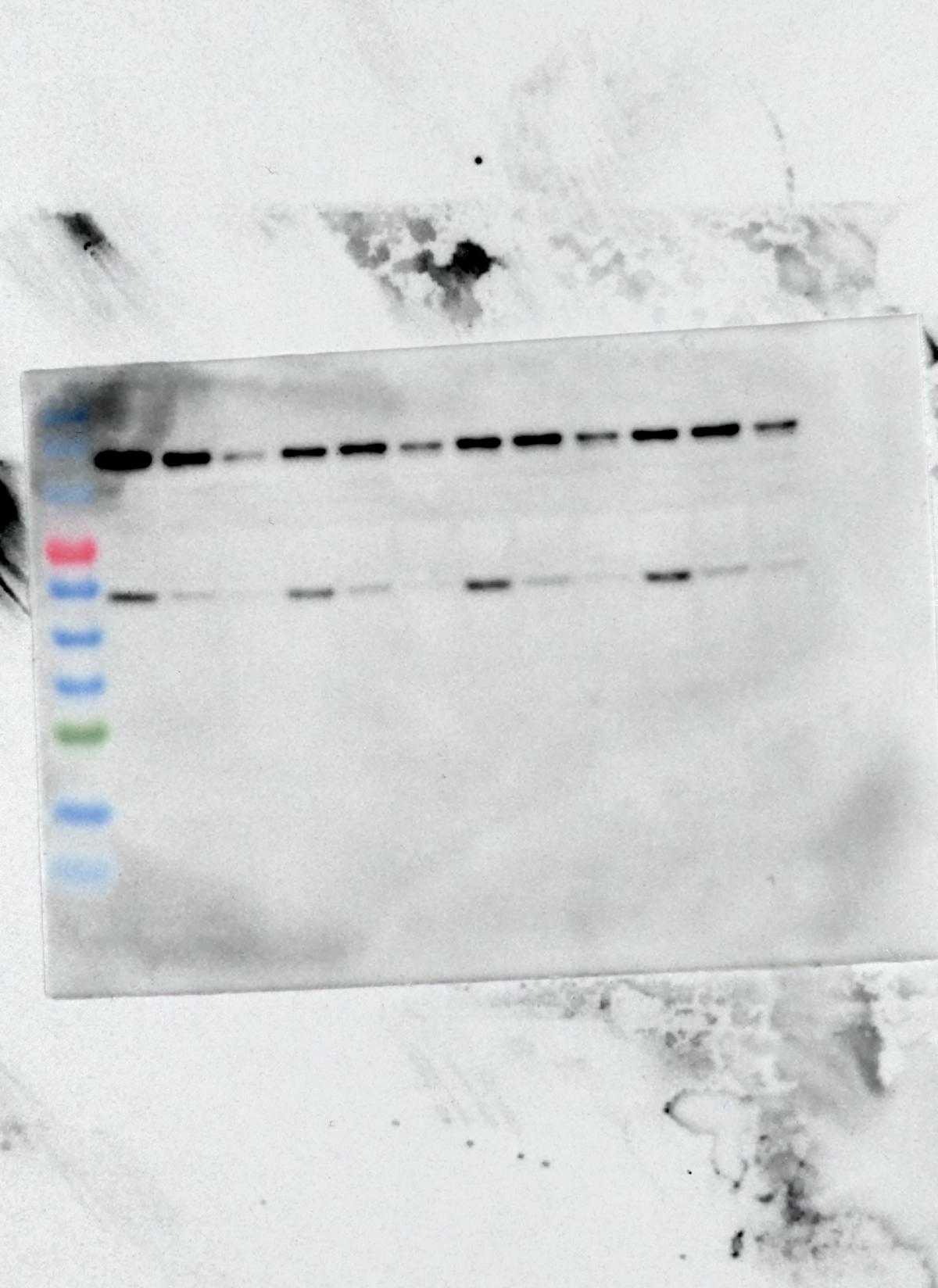

Supplement: Supplementary file 1 [file biology-11-01761-s001.zip › File S2/P-IRE1/p,ire1 2.jpg]

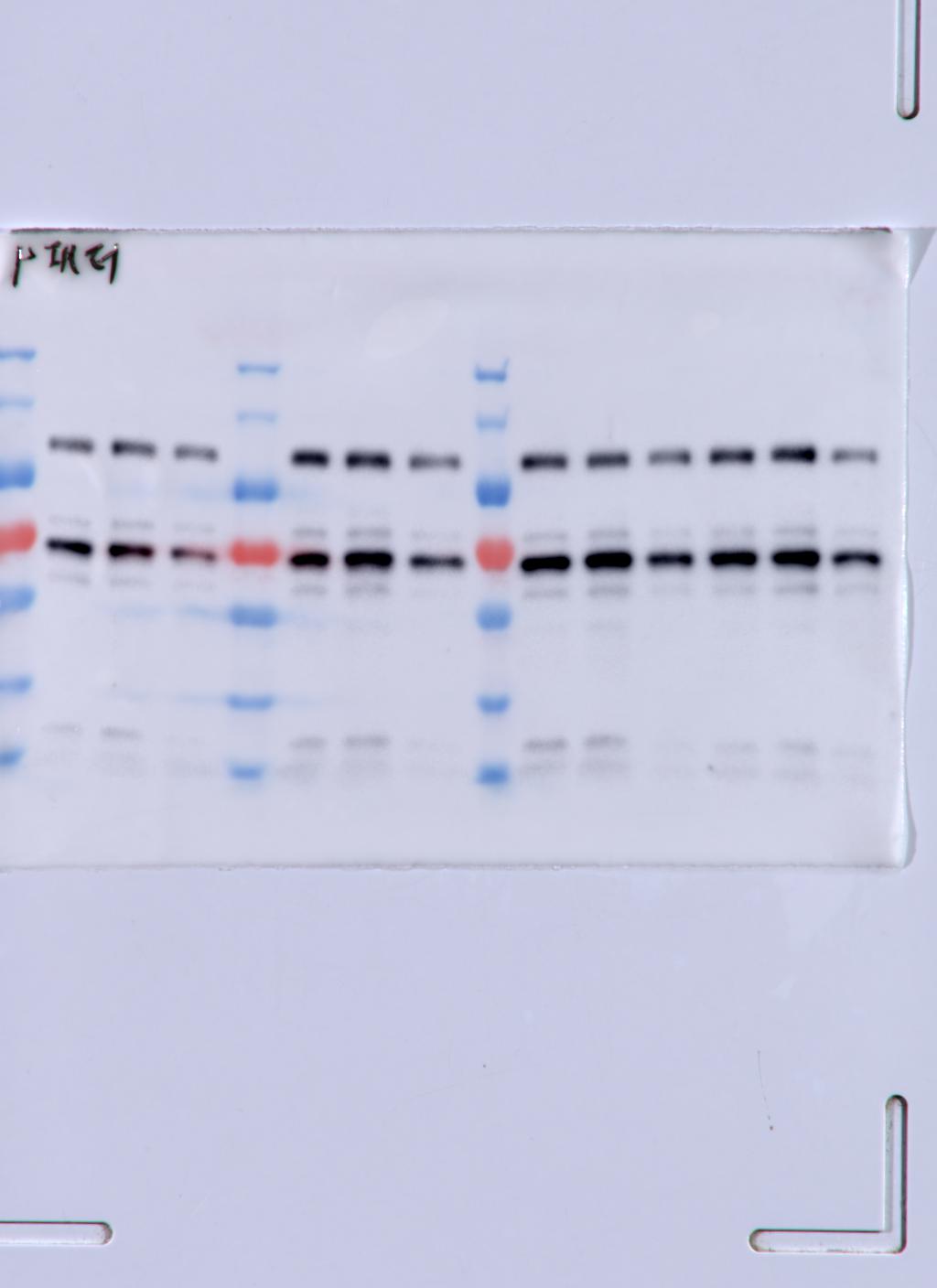

Supplement: Supplementary file 1 [file biology-11-01761-s001.zip › File S2/P-IRE1/pire1 1.jpg]

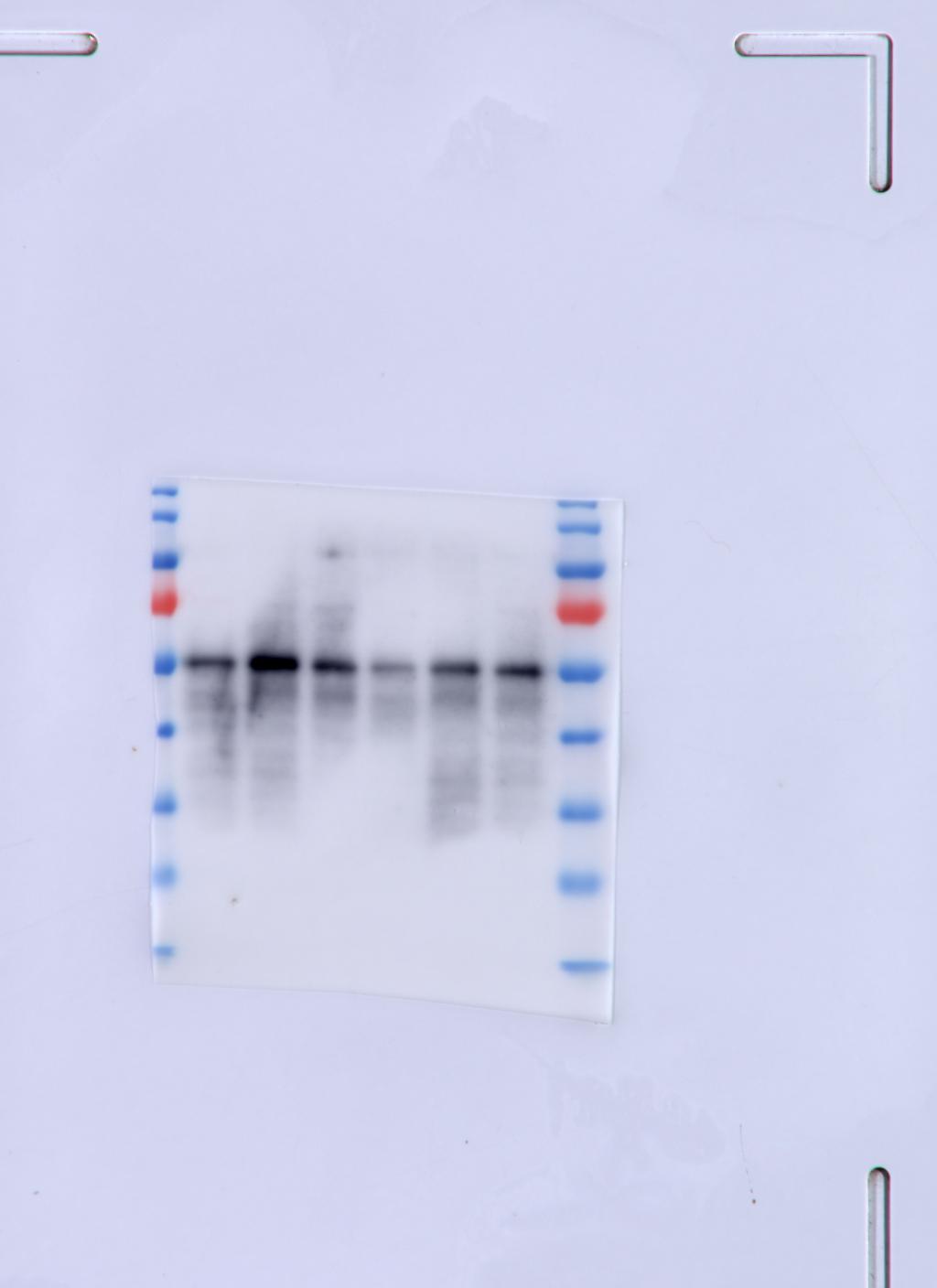

Supplement: Supplementary file 1 [file biology-11-01761-s001.zip › File S2/TXNIP/txnip 2022.06.18_15.05.41_Ch+Marker.jpg]

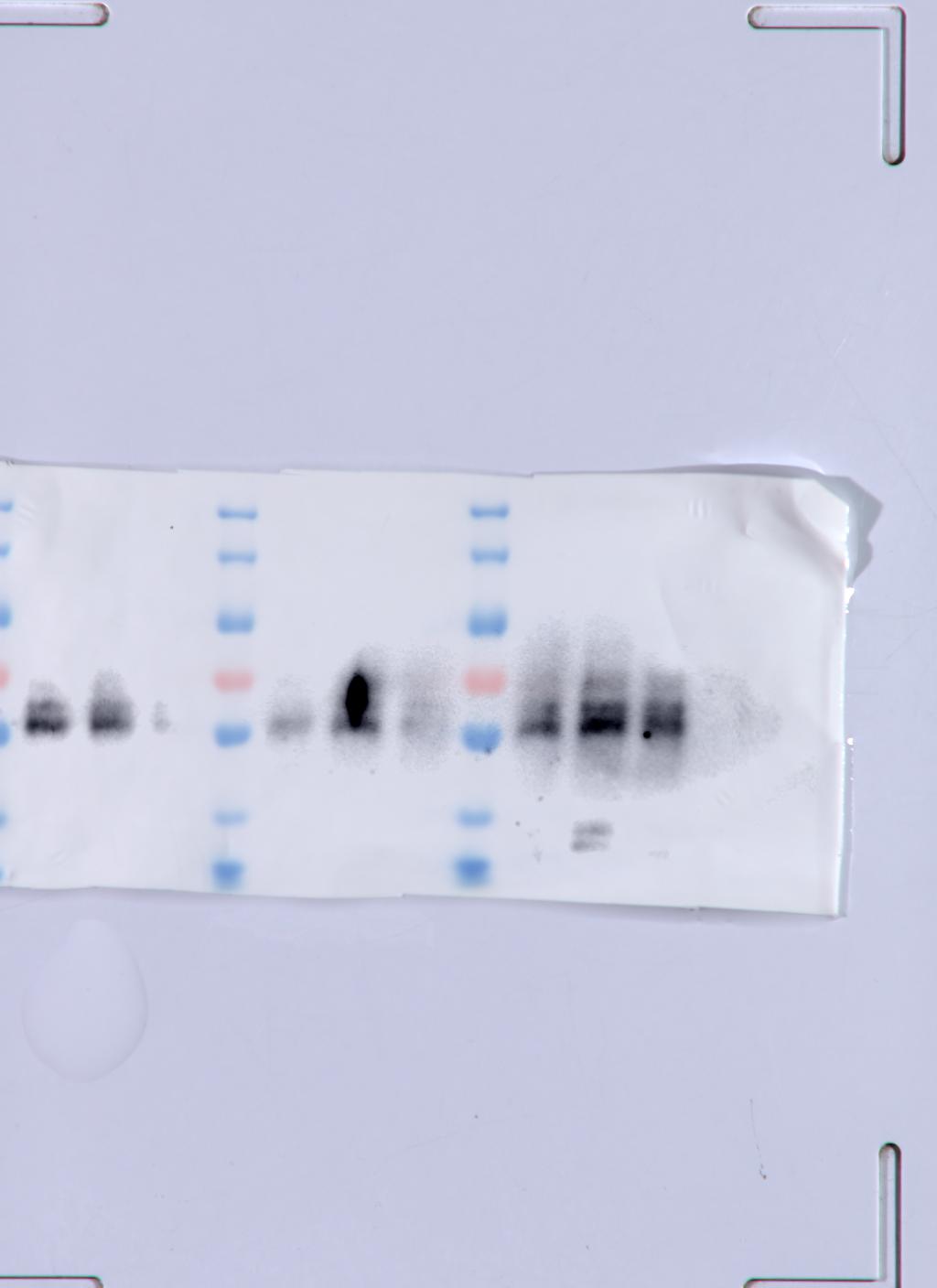

Supplement: Supplementary file 1 [file biology-11-01761-s001.zip › File S2/TXNIP/txnip2022.03.29_14.35.50_Ch+Marker.jpg]

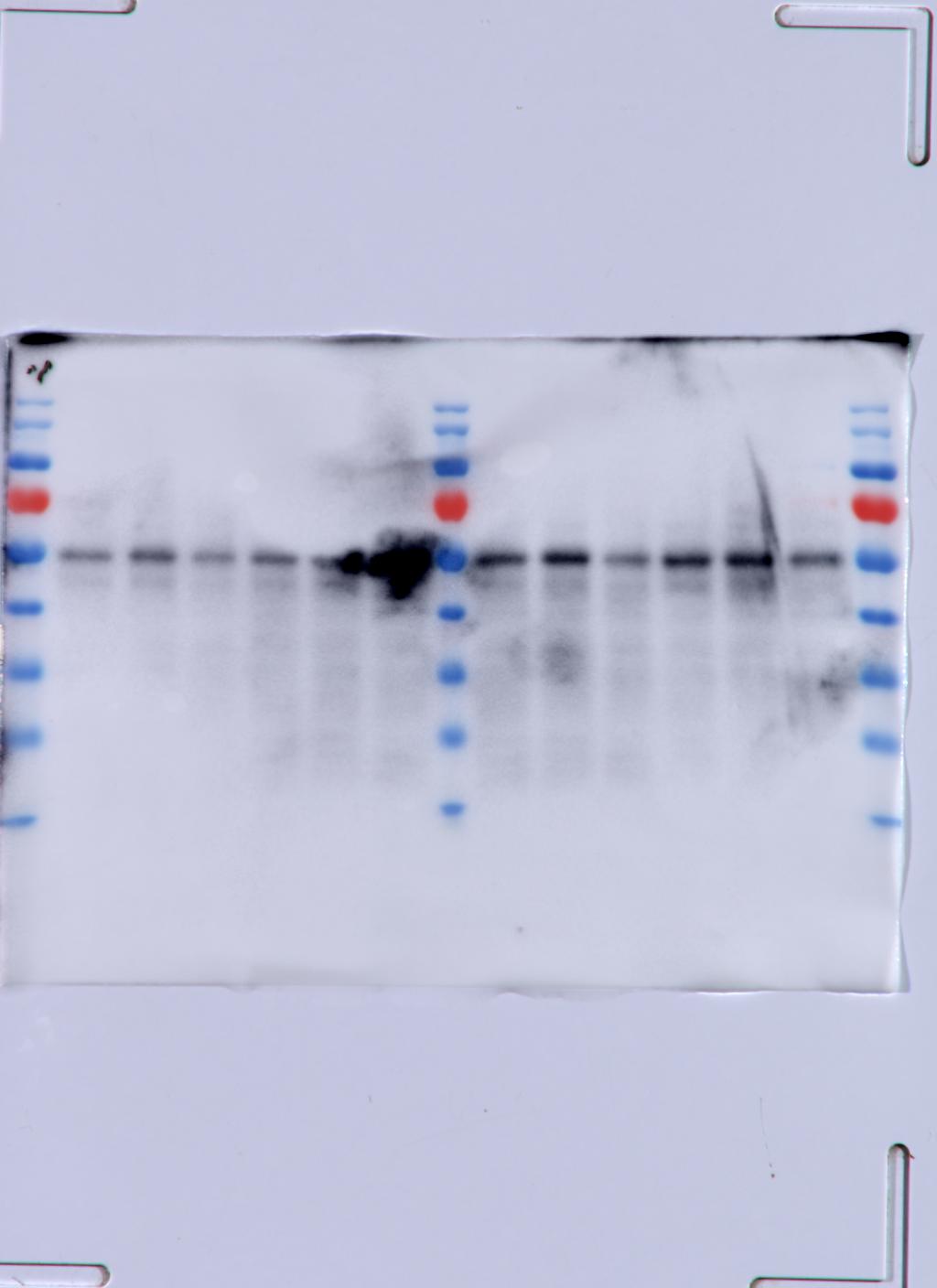

Supplement: Supplementary file 1 [file biology-11-01761-s001.zip › File S2/TXNIP/ztxnip 2022.06.12_20.34.11_Ch+Marker.jpg]

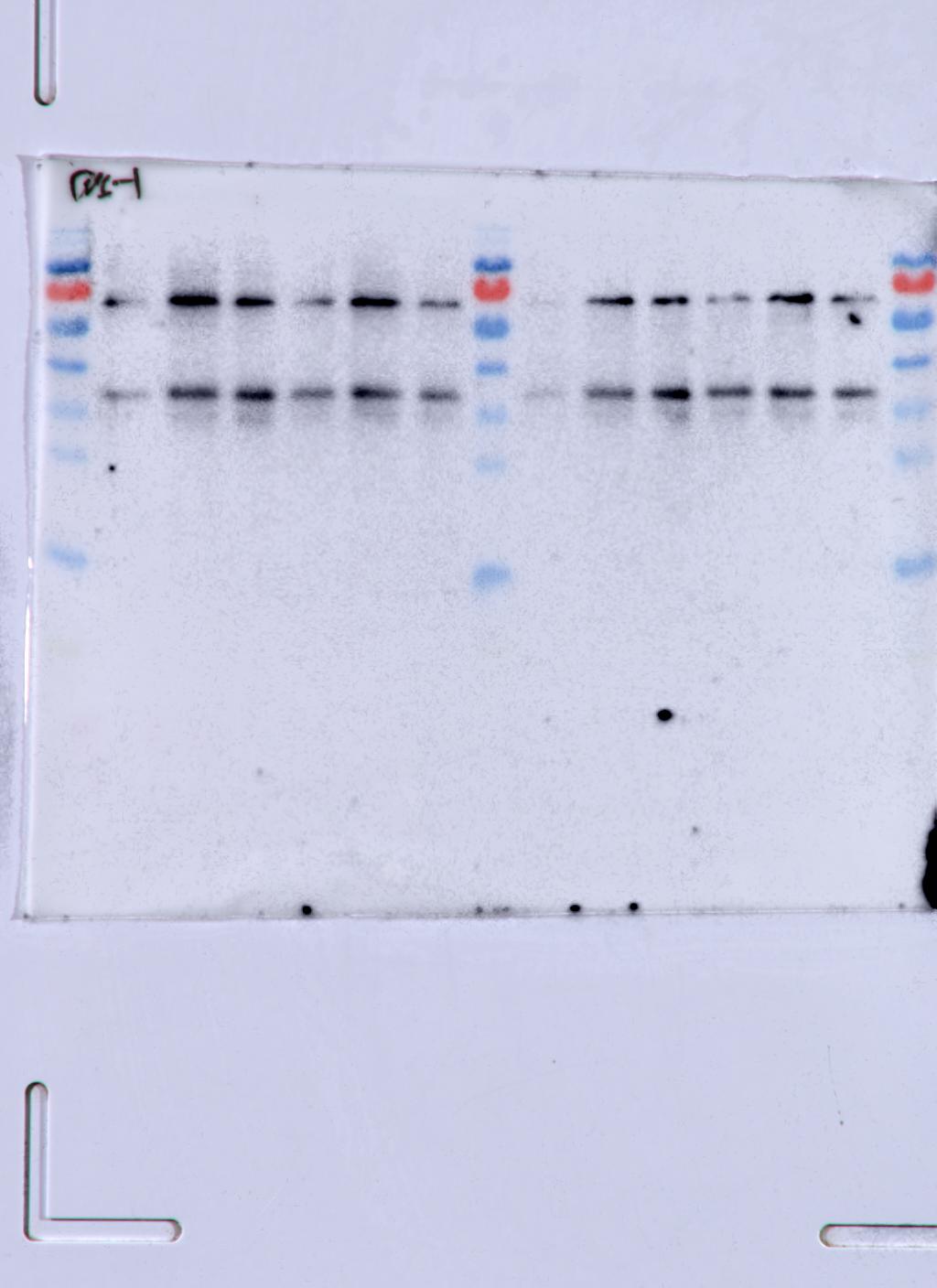

Supplement: Supplementary file 1 [file biology-11-01761-s001.zip › File S3/The data related to the effect of ciproterone acetate on the alleviation of ovarian granulosa cell pyroptosis and IRE1a┴ pathway activation in vitro/Cleave caspase-1andcaspase-1/cas1 2022.03.29_14.44.29_Ch+Marker.jpg]

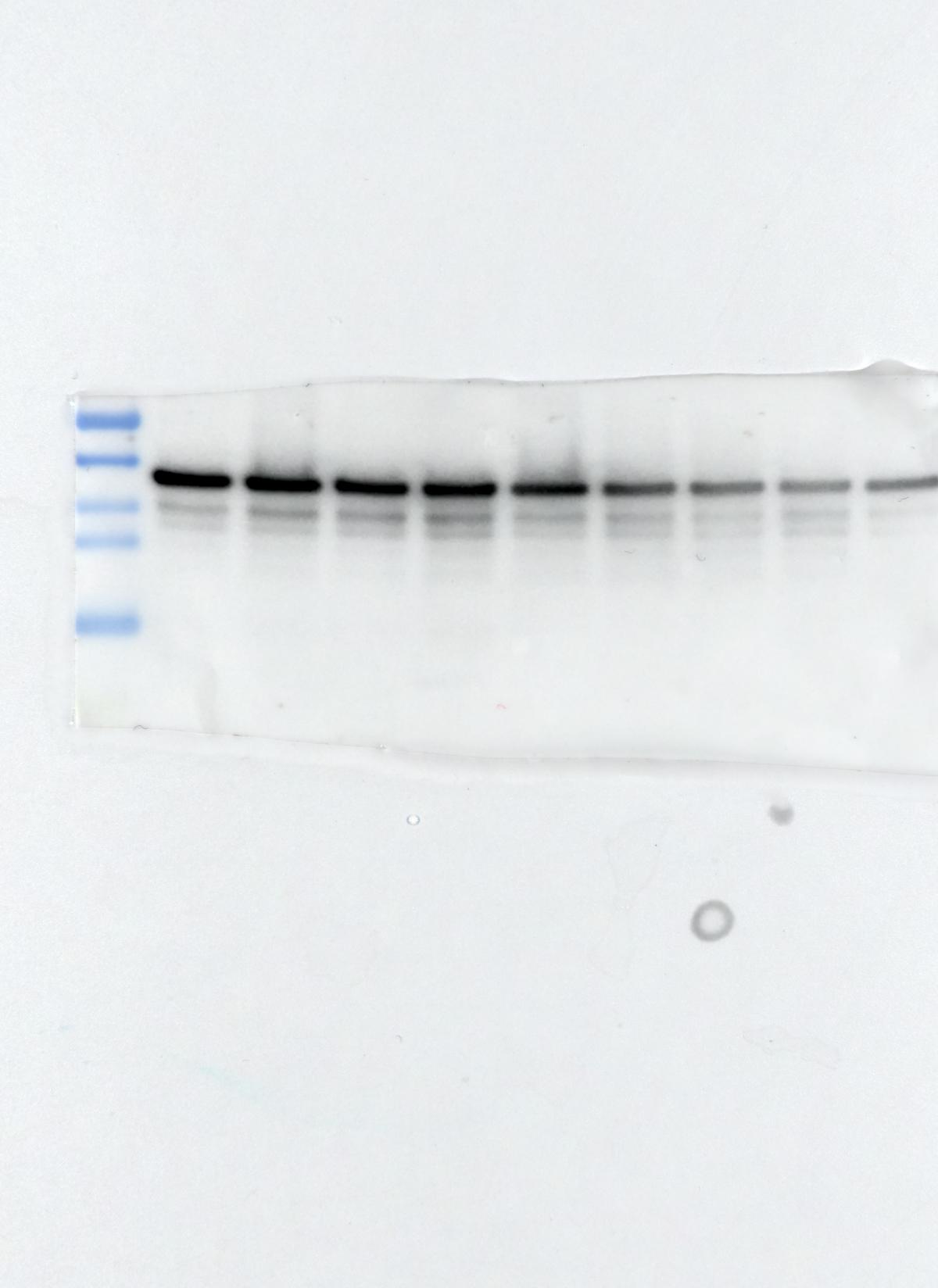

Supplement: Supplementary file 1 [file biology-11-01761-s001.zip › File S3/The data related to the effect of ciproterone acetate on the alleviation of ovarian granulosa cell pyroptosis and IRE1a┴ pathway activation in vitro/GAPDH/gapdh.10.jpg]

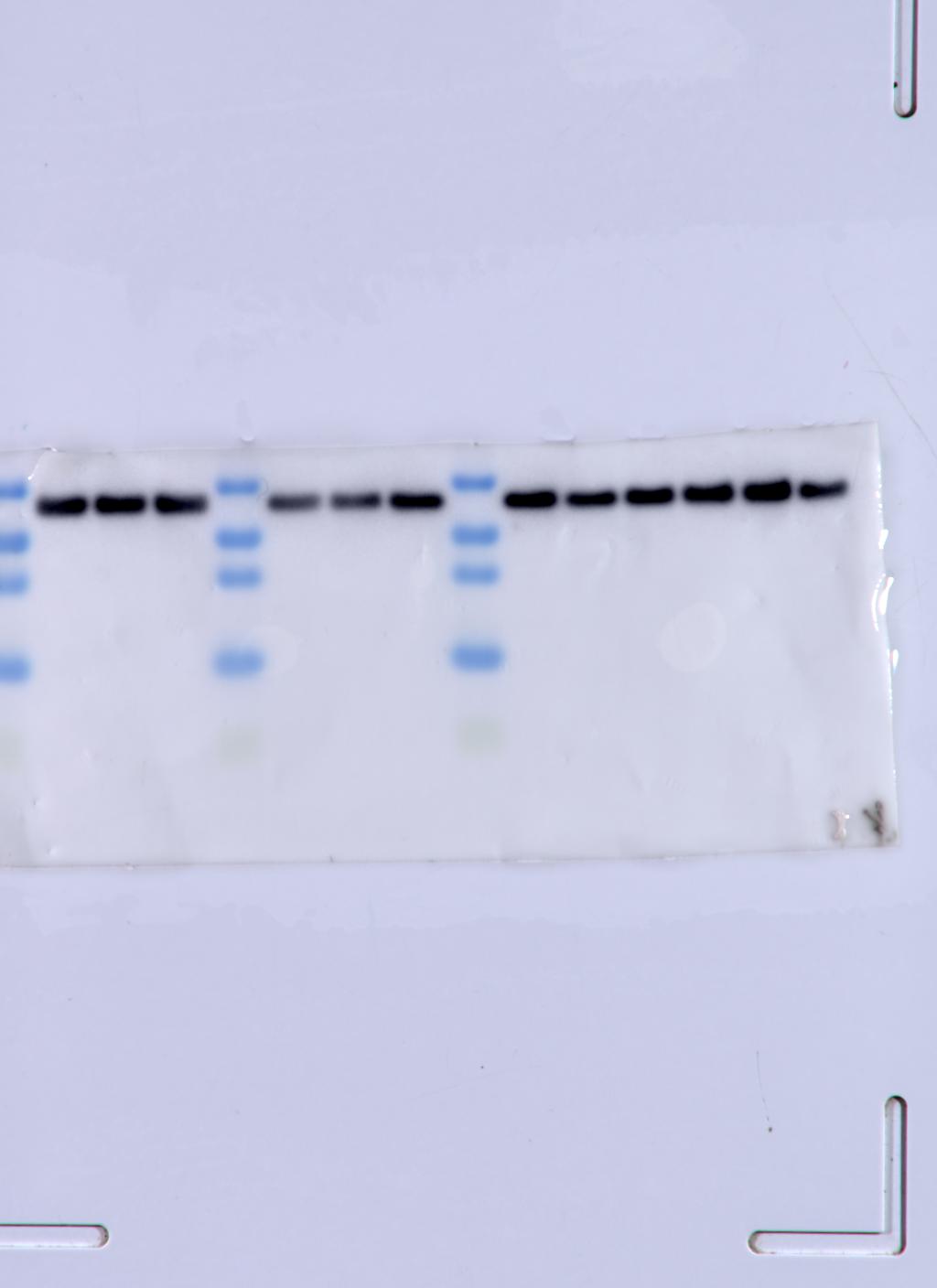

Supplement: Supplementary file 1 [file biology-11-01761-s001.zip › File S3/The data related to the effect of ciproterone acetate on the alleviation of ovarian granulosa cell pyroptosis and IRE1a┴ pathway activation in vitro/GAPDH/GAPDH15.jpg]

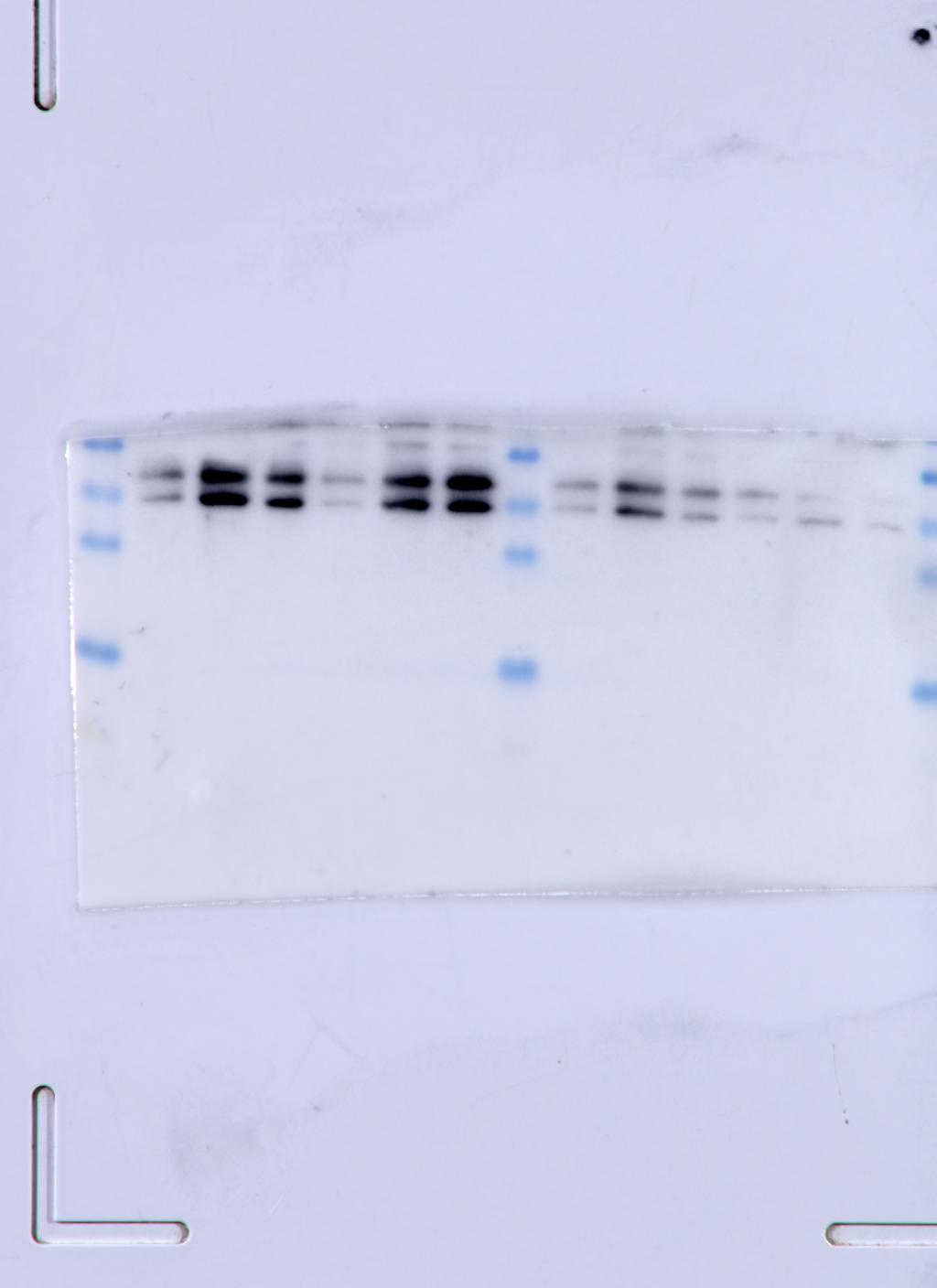

Supplement: Supplementary file 1 [file biology-11-01761-s001.zip › File S3/The data related to the effect of ciproterone acetate on the alleviation of ovarian granulosa cell pyroptosis and IRE1a┴ pathway activation in vitro/GD-N/gd-n.jpg]

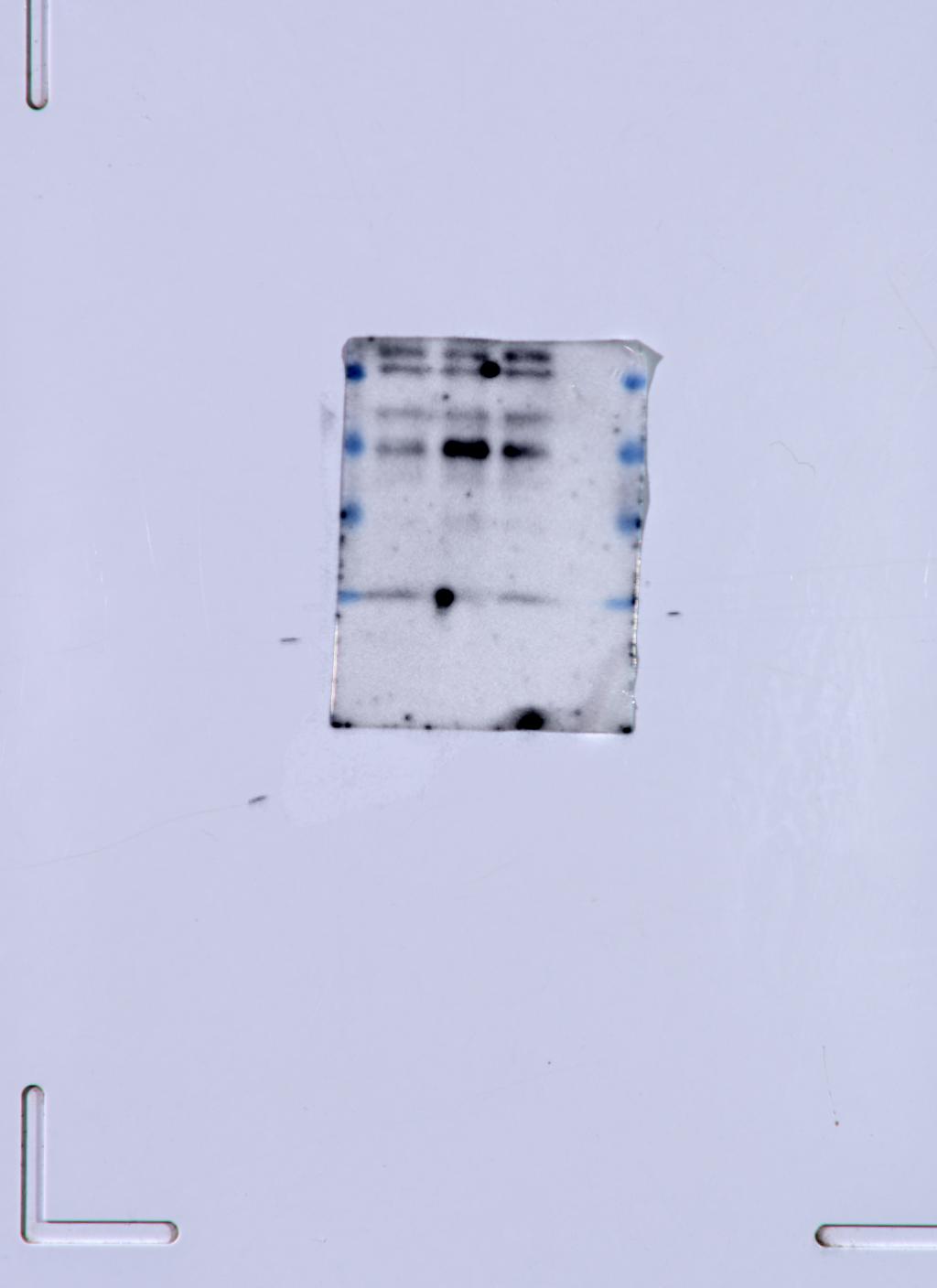

Supplement: Supplementary file 1 [file biology-11-01761-s001.zip › File S3/The data related to the effect of ciproterone acetate on the alleviation of ovarian granulosa cell pyroptosis and IRE1a┴ pathway activation in vitro/GD-N/gdn2.jpg]

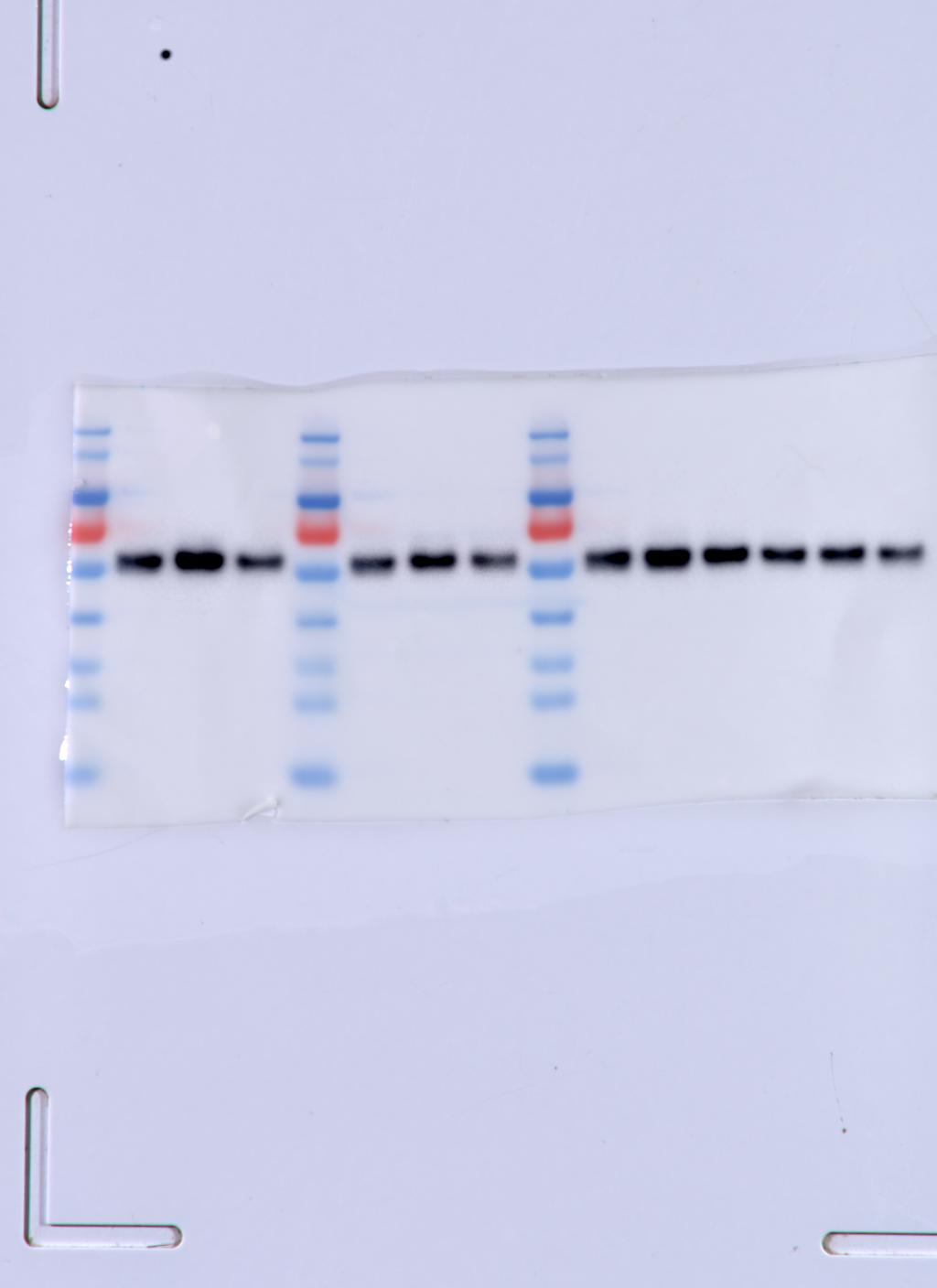

Supplement: Supplementary file 1 [file biology-11-01761-s001.zip › File S3/The data related to the effect of ciproterone acetate on the alleviation of ovarian granulosa cell pyroptosis and IRE1a┴ pathway activation in vitro/GD/gd.jpg]

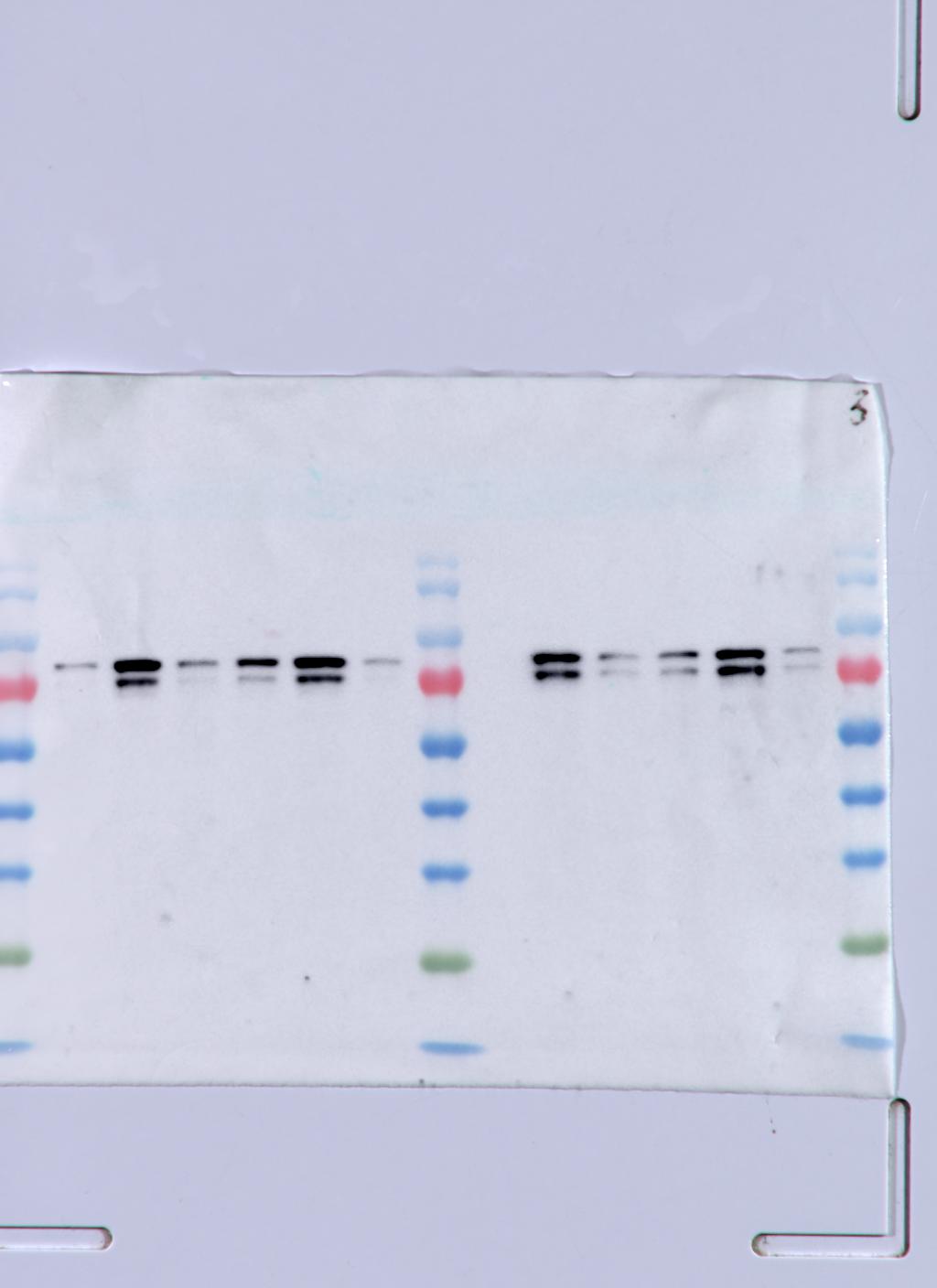

Supplement: Supplementary file 1 [file biology-11-01761-s001.zip › File S3/The data related to the effect of ciproterone acetate on the alleviation of ovarian granulosa cell pyroptosis and IRE1a┴ pathway activation in vitro/GRP78/781 2022.05.23_13.51.31_Ch+Marker.jpg]

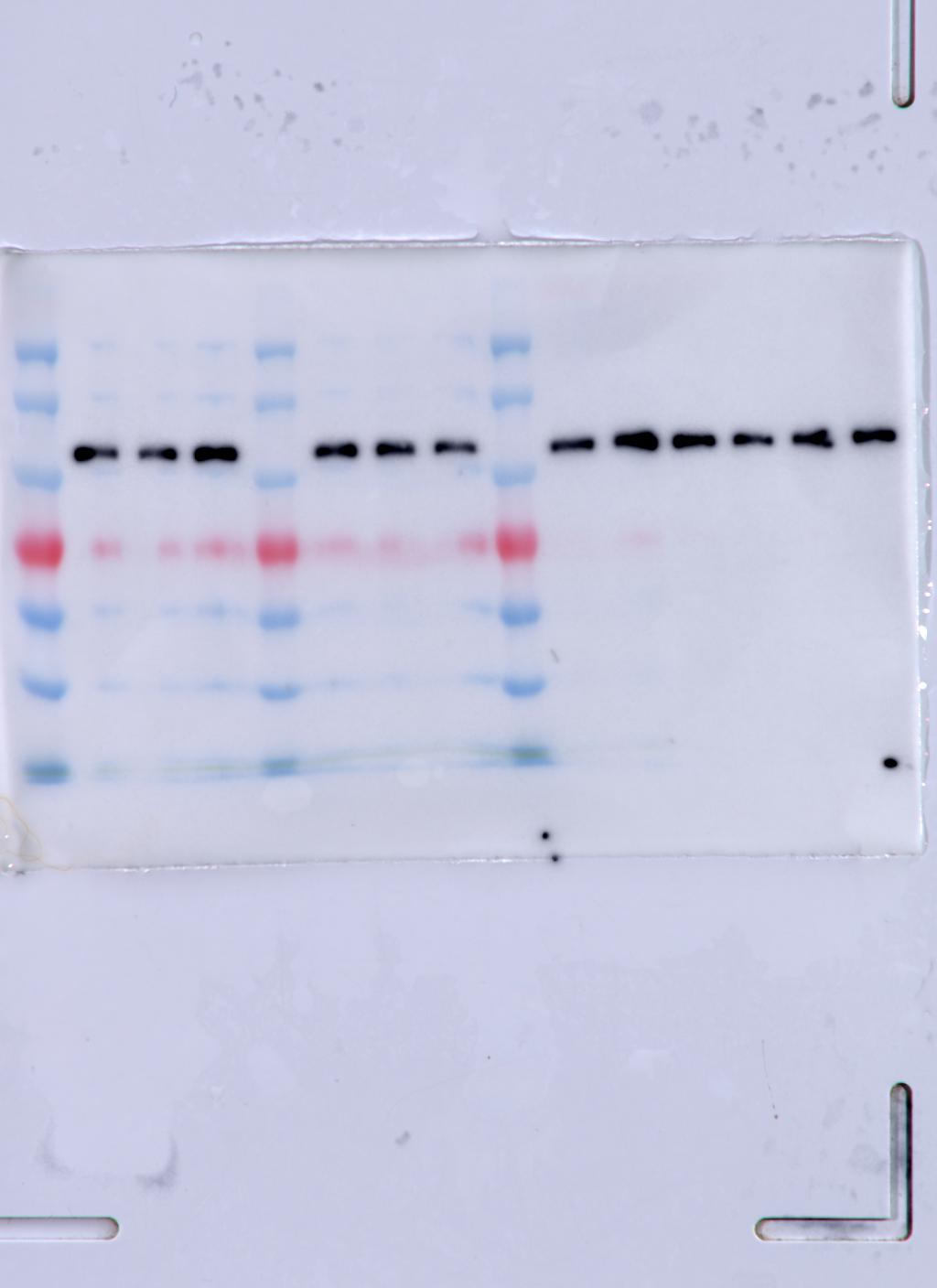

Supplement: Supplementary file 1 [file biology-11-01761-s001.zip › File S3/The data related to the effect of ciproterone acetate on the alleviation of ovarian granulosa cell pyroptosis and IRE1a┴ pathway activation in vitro/IRE1/ire1.jpg]

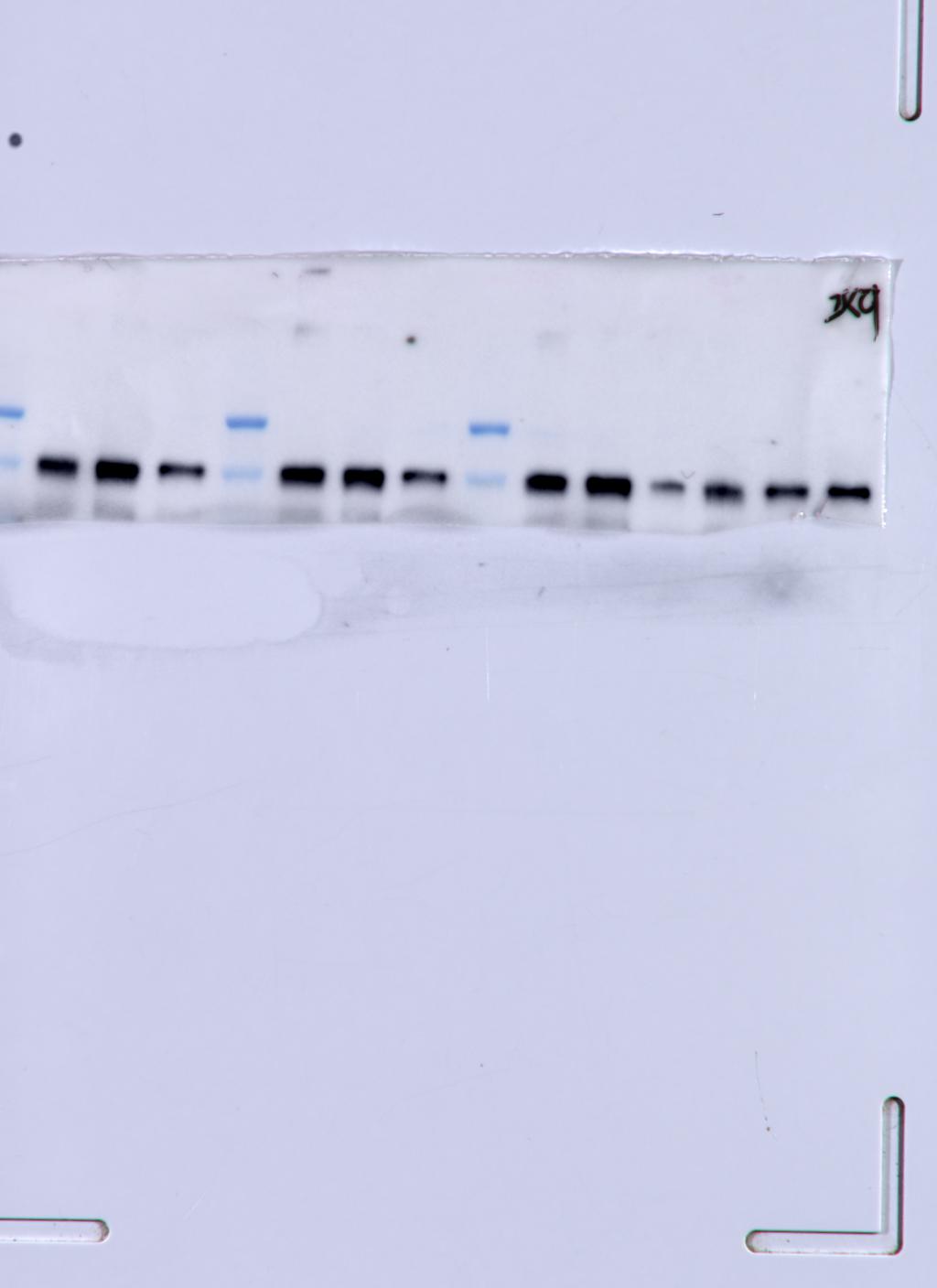

Supplement: Supplementary file 1 [file biology-11-01761-s001.zip › File S3/The data related to the effect of ciproterone acetate on the alleviation of ovarian granulosa cell pyroptosis and IRE1a┴ pathway activation in vitro/IRE1/ire2.jpg]

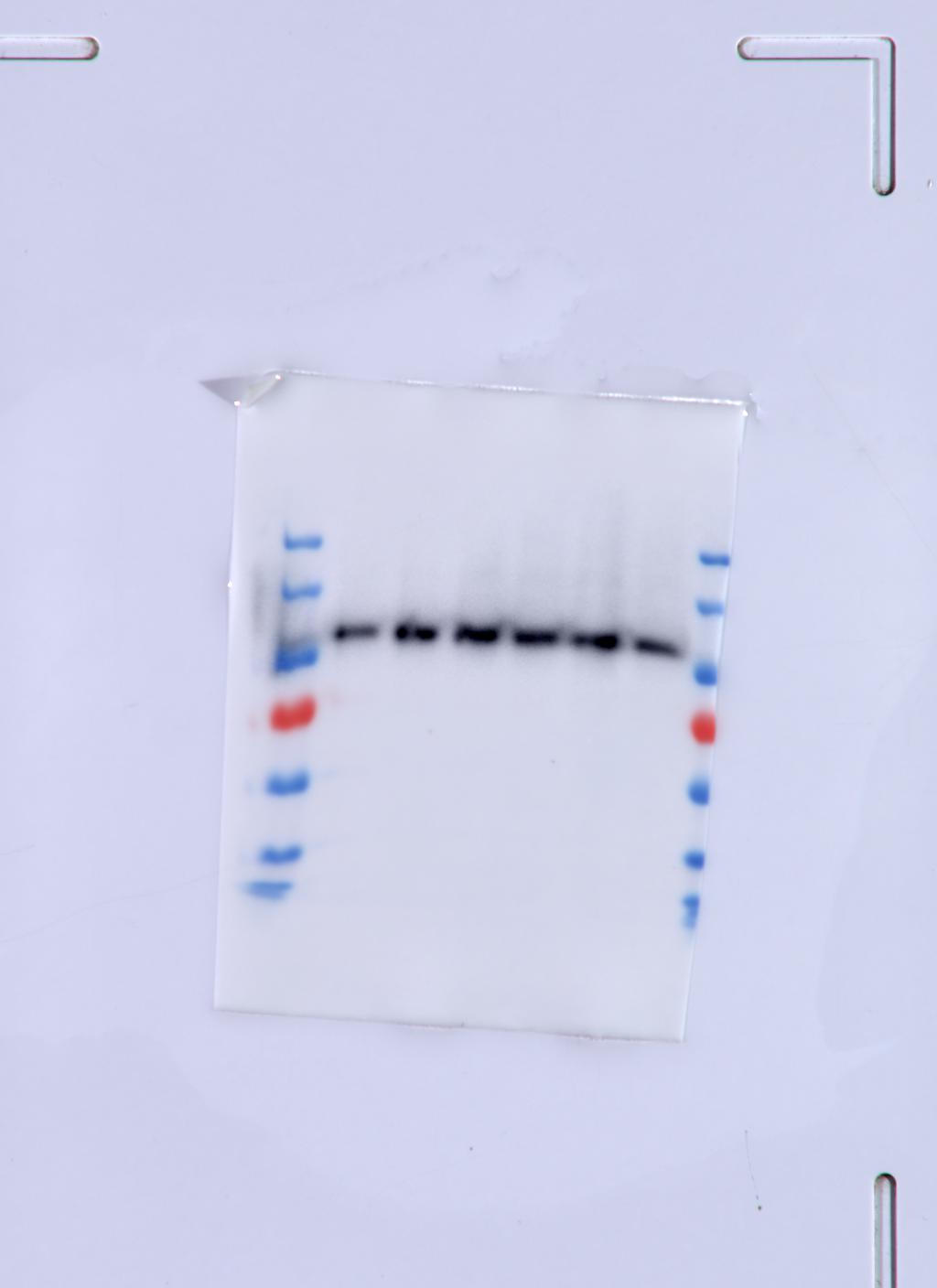

Supplement: Supplementary file 1 [file biology-11-01761-s001.zip › File S3/The data related to the effect of ciproterone acetate on the alleviation of ovarian granulosa cell pyroptosis and IRE1a┴ pathway activation in vitro/NLRP3/nlrp3 1 .jpg]

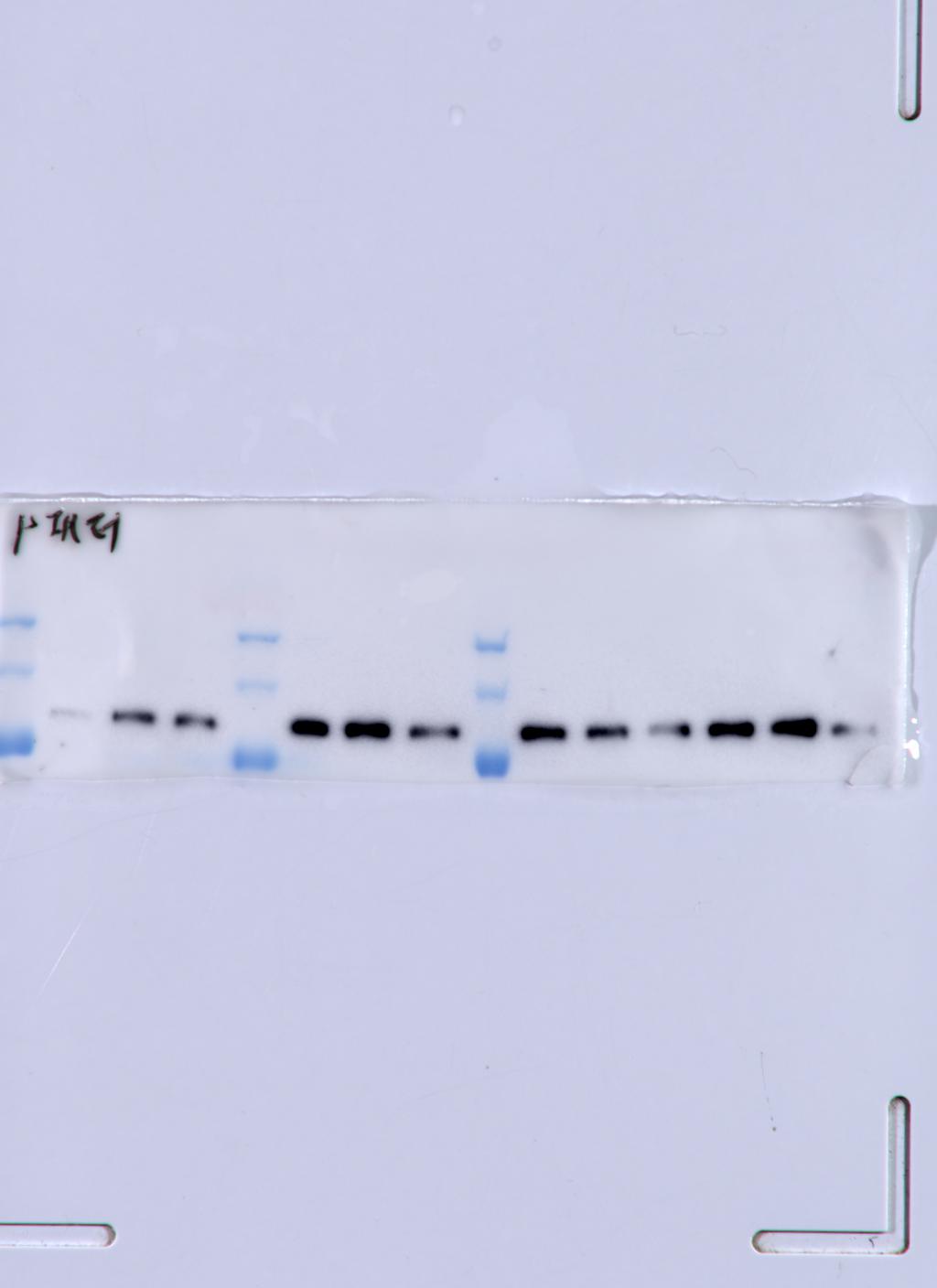

Supplement: Supplementary file 1 [file biology-11-01761-s001.zip › File S3/The data related to the effect of ciproterone acetate on the alleviation of ovarian granulosa cell pyroptosis and IRE1a┴ pathway activation in vitro/P-IRE1/pire1.jpg]

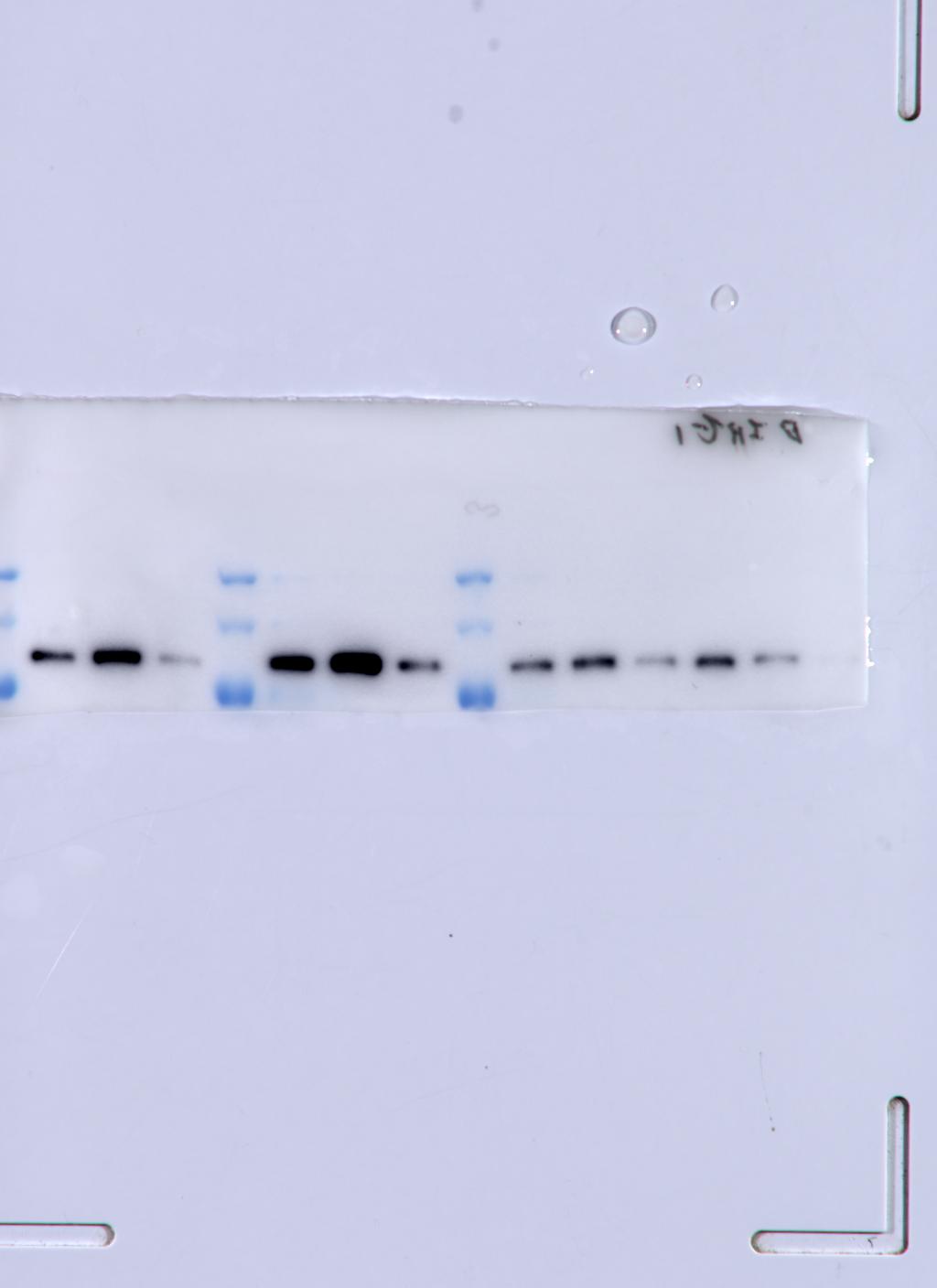

Supplement: Supplementary file 1 [file biology-11-01761-s001.zip › File S3/The data related to the effect of ciproterone acetate on the alleviation of ovarian granulosa cell pyroptosis and IRE1a┴ pathway activation in vitro/P-IRE1/pire2.jpg]

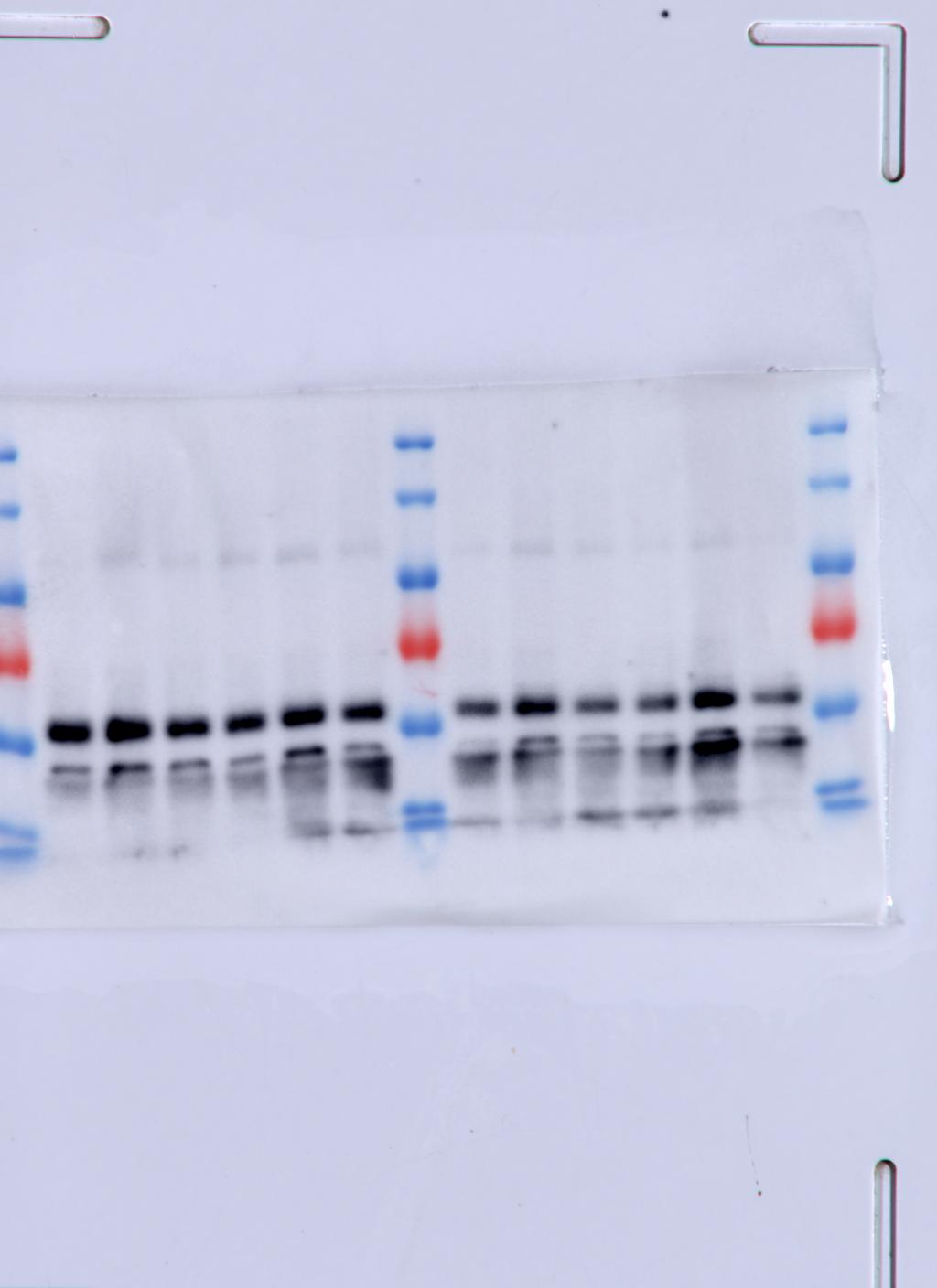

Supplement: Supplementary file 1 [file biology-11-01761-s001.zip › File S3/The data related to the effect of ciproterone acetate on the alleviation of ovarian granulosa cell pyroptosis and IRE1a┴ pathway activation in vitro/TXNIP/txnip 2022.09.22_20.41.10_Ch+Marker.jpg]

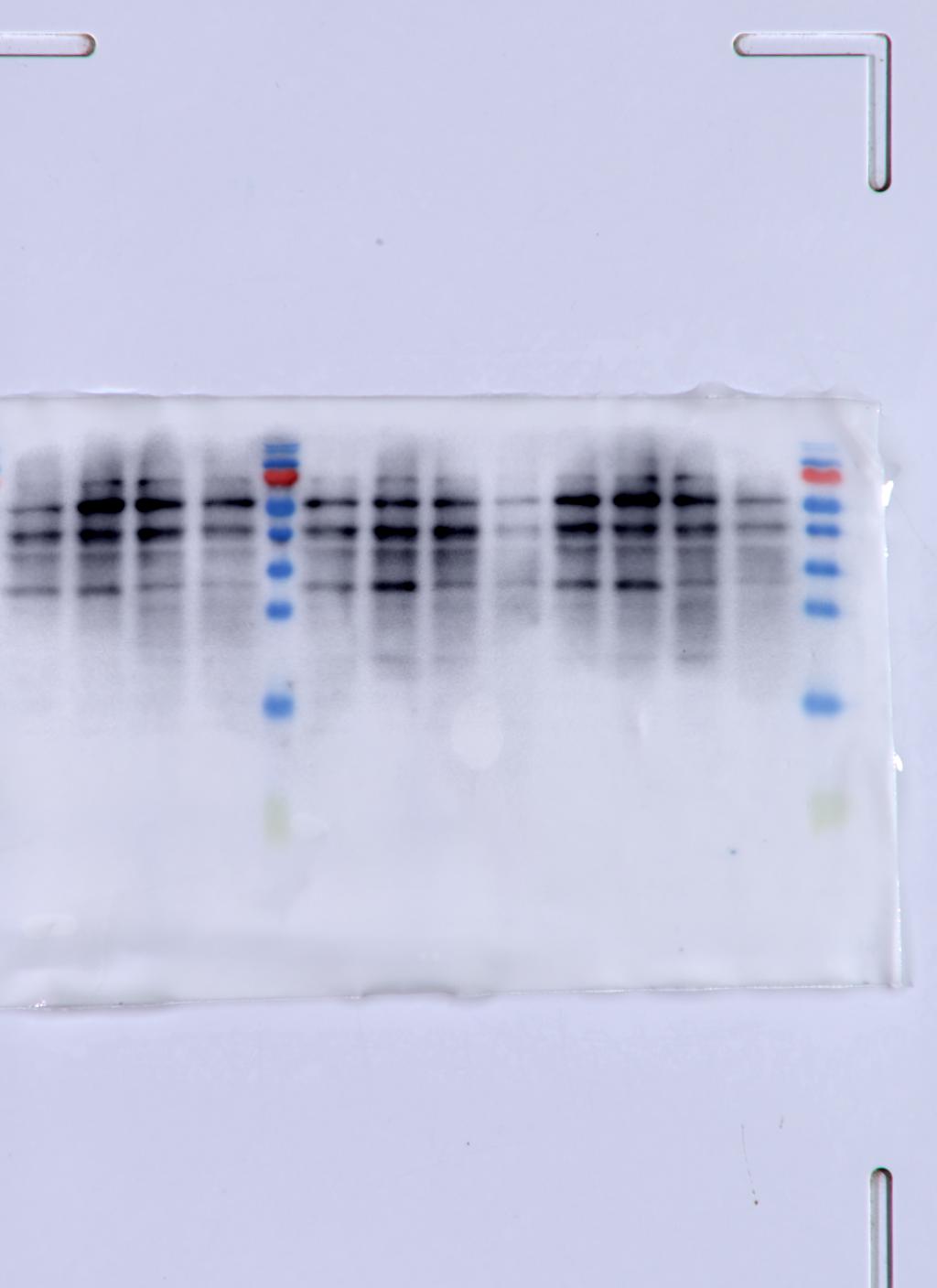

Supplement: Supplementary file 1 [file biology-11-01761-s001.zip › File S3/the relevant data of the vitro experiment to explore the effect and mechanism of cyproterone acetate on ovarian granulosa cell pyroptosis/cleave caspase-1and caspase-1/c-cas.jpg]

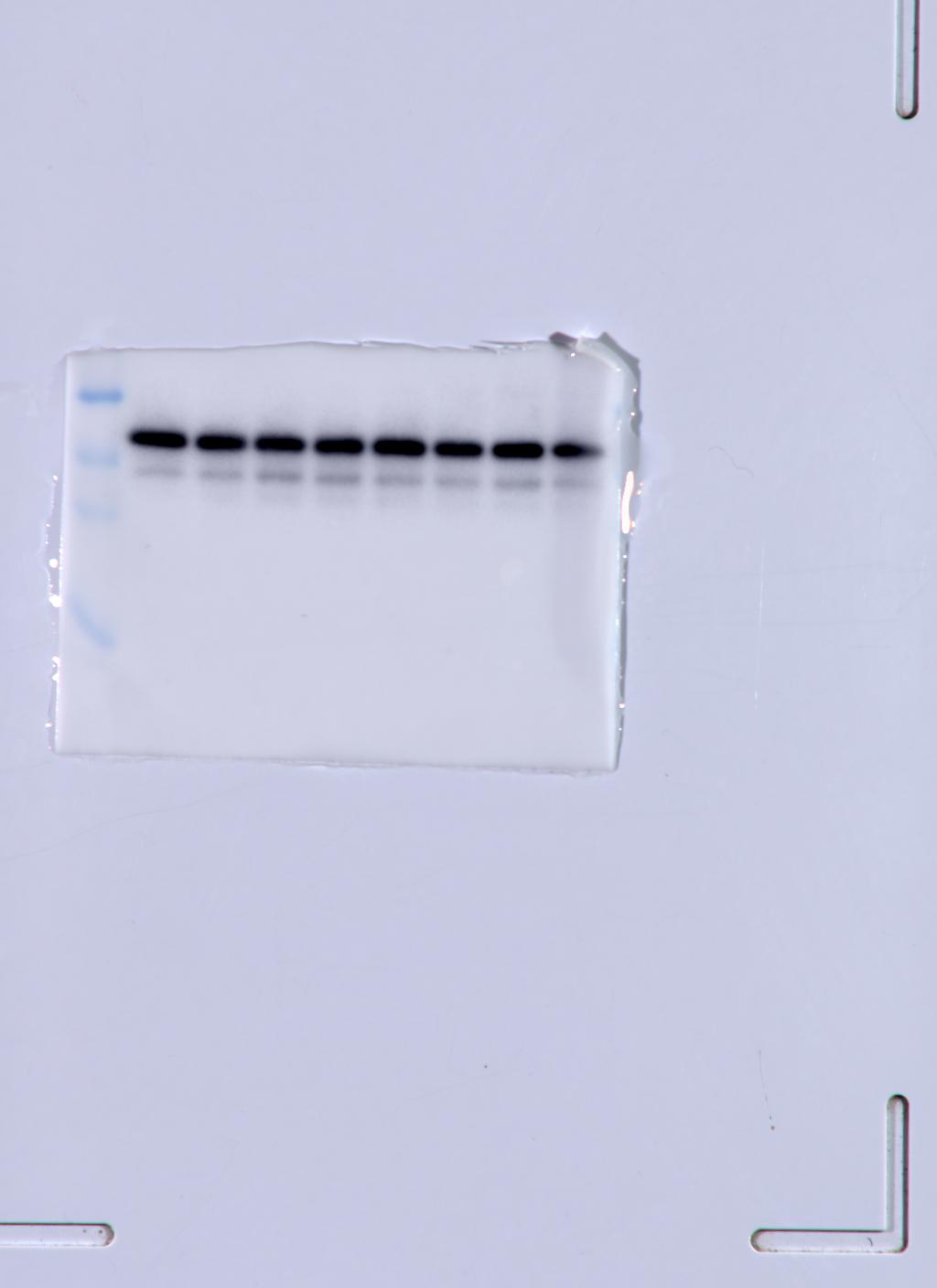

Supplement: Supplementary file 1 [file biology-11-01761-s001.zip › File S3/the relevant data of the vitro experiment to explore the effect and mechanism of cyproterone acetate on ovarian granulosa cell pyroptosis/GAPDH.jpg]

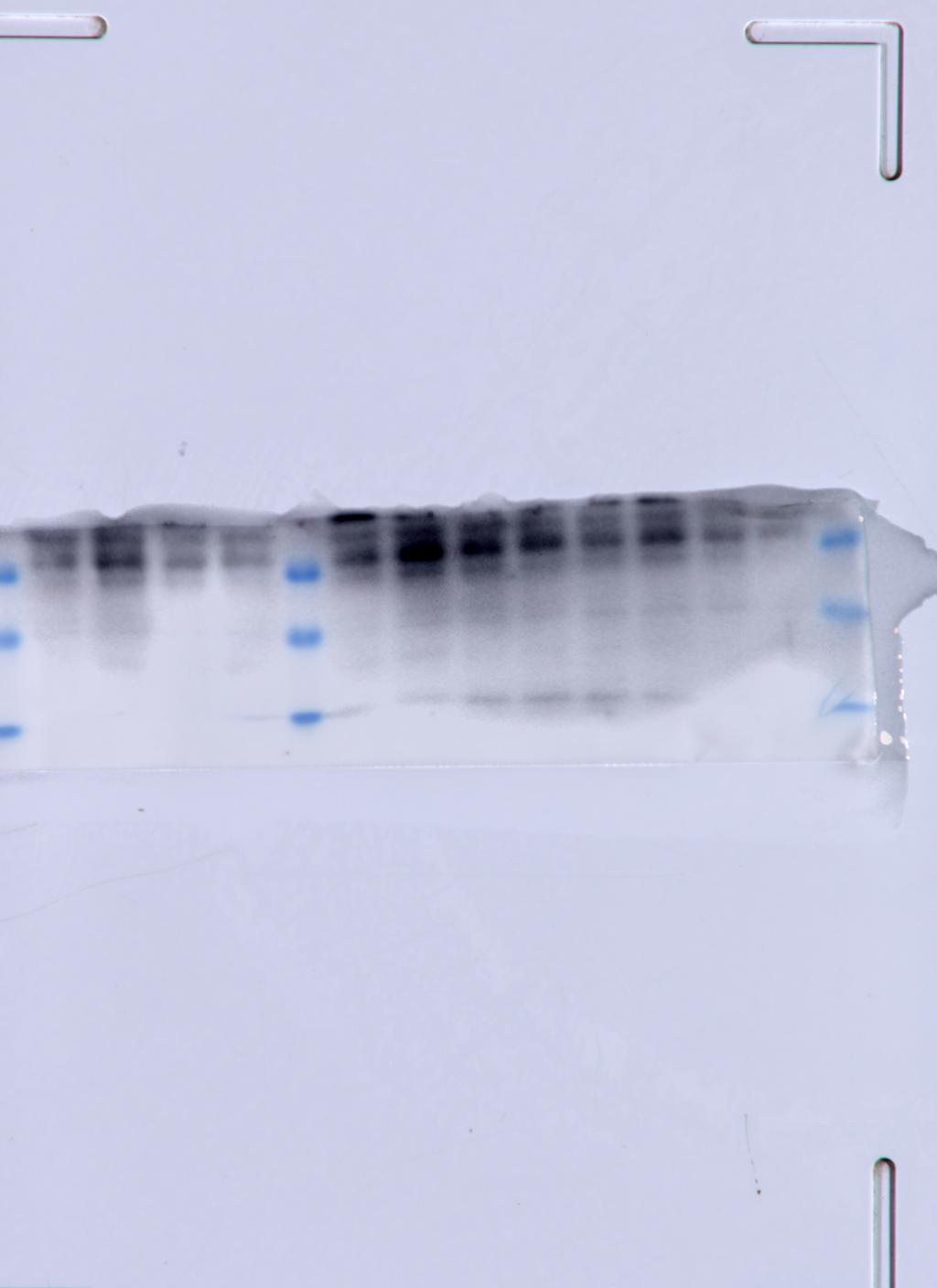

Supplement: Supplementary file 1 [file biology-11-01761-s001.zip › File S3/the relevant data of the vitro experiment to explore the effect and mechanism of cyproterone acetate on ovarian granulosa cell pyroptosis/GD-N/GD-N.jpg]

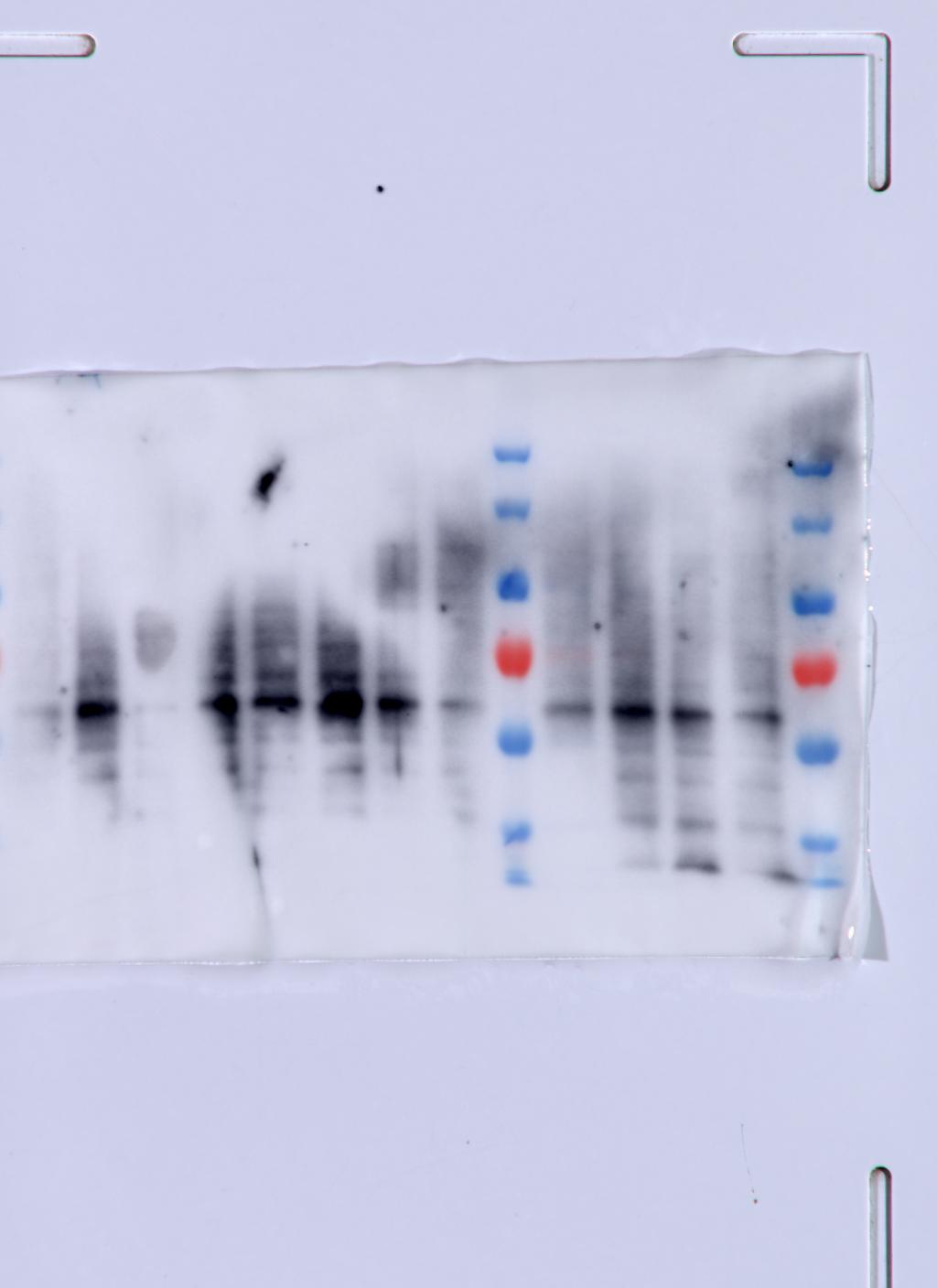

Supplement: Supplementary file 1 [file biology-11-01761-s001.zip › File S3/the relevant data of the vitro experiment to explore the effect and mechanism of cyproterone acetate on ovarian granulosa cell pyroptosis/GD/gd 1.jpg]

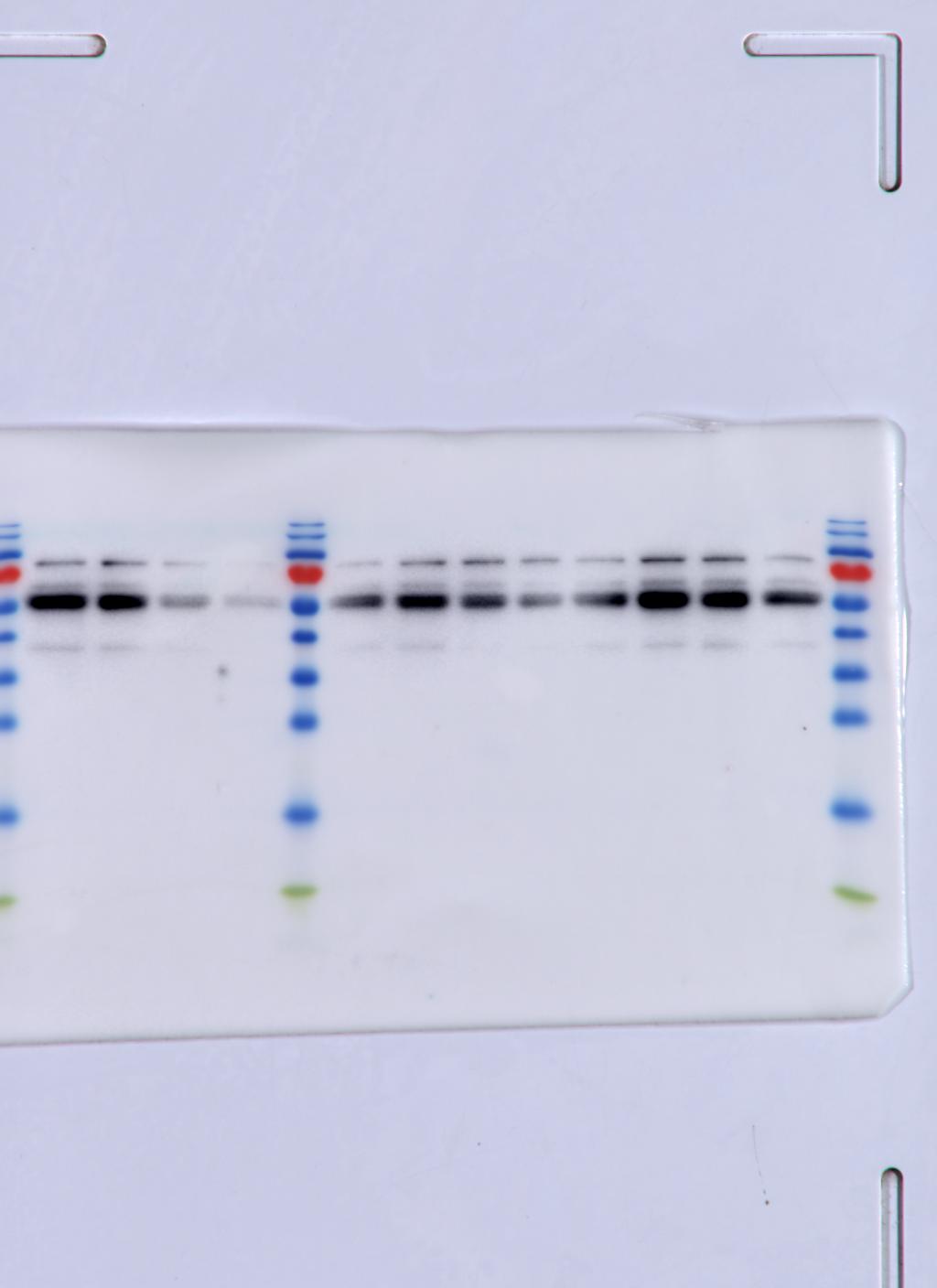

Supplement: Supplementary file 1 [file biology-11-01761-s001.zip › File S3/the relevant data of the vitro experiment to explore the effect and mechanism of cyproterone acetate on ovarian granulosa cell pyroptosis/GD/gd2.jpg]

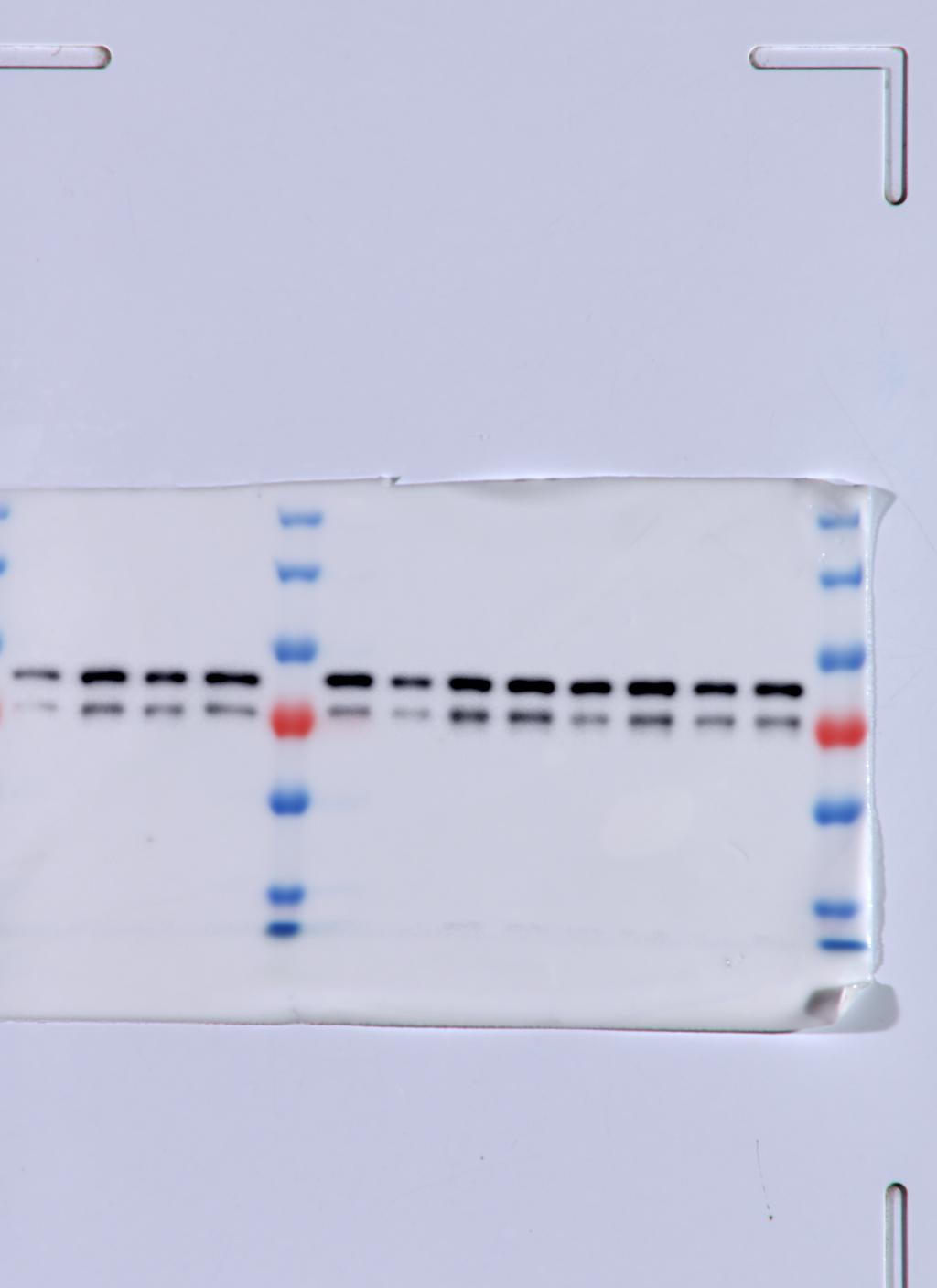

Supplement: Supplementary file 1 [file biology-11-01761-s001.zip › File S3/the relevant data of the vitro experiment to explore the effect and mechanism of cyproterone acetate on ovarian granulosa cell pyroptosis/grp78/78ok2022.09.14_20.11.27_Ch+Marker.jpg]

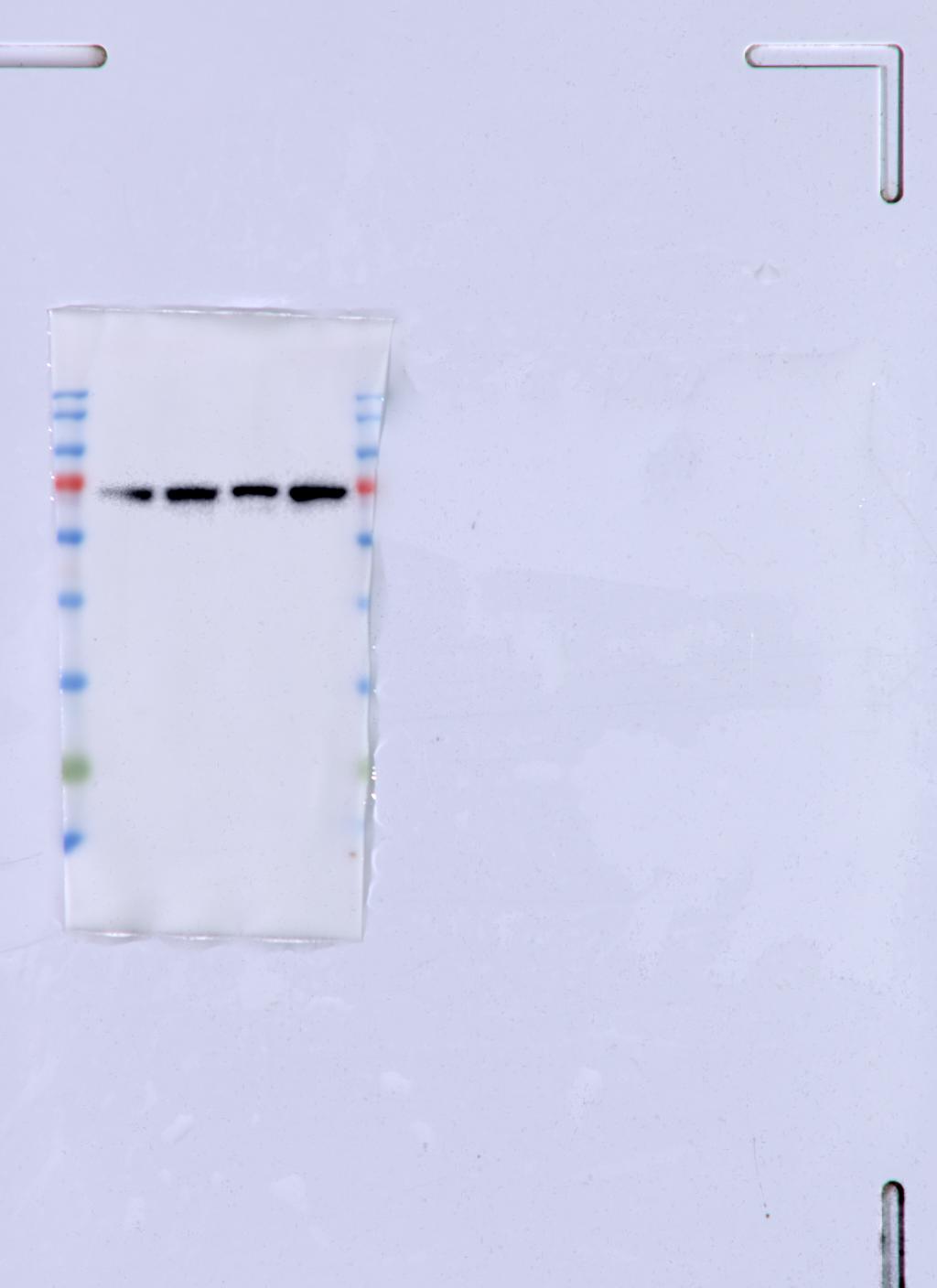

Supplement: Supplementary file 1 [file biology-11-01761-s001.zip › File S3/the relevant data of the vitro experiment to explore the effect and mechanism of cyproterone acetate on ovarian granulosa cell pyroptosis/grp78/grp78111 2022.07.18_20.05.29_Ch+Marker.jpg]

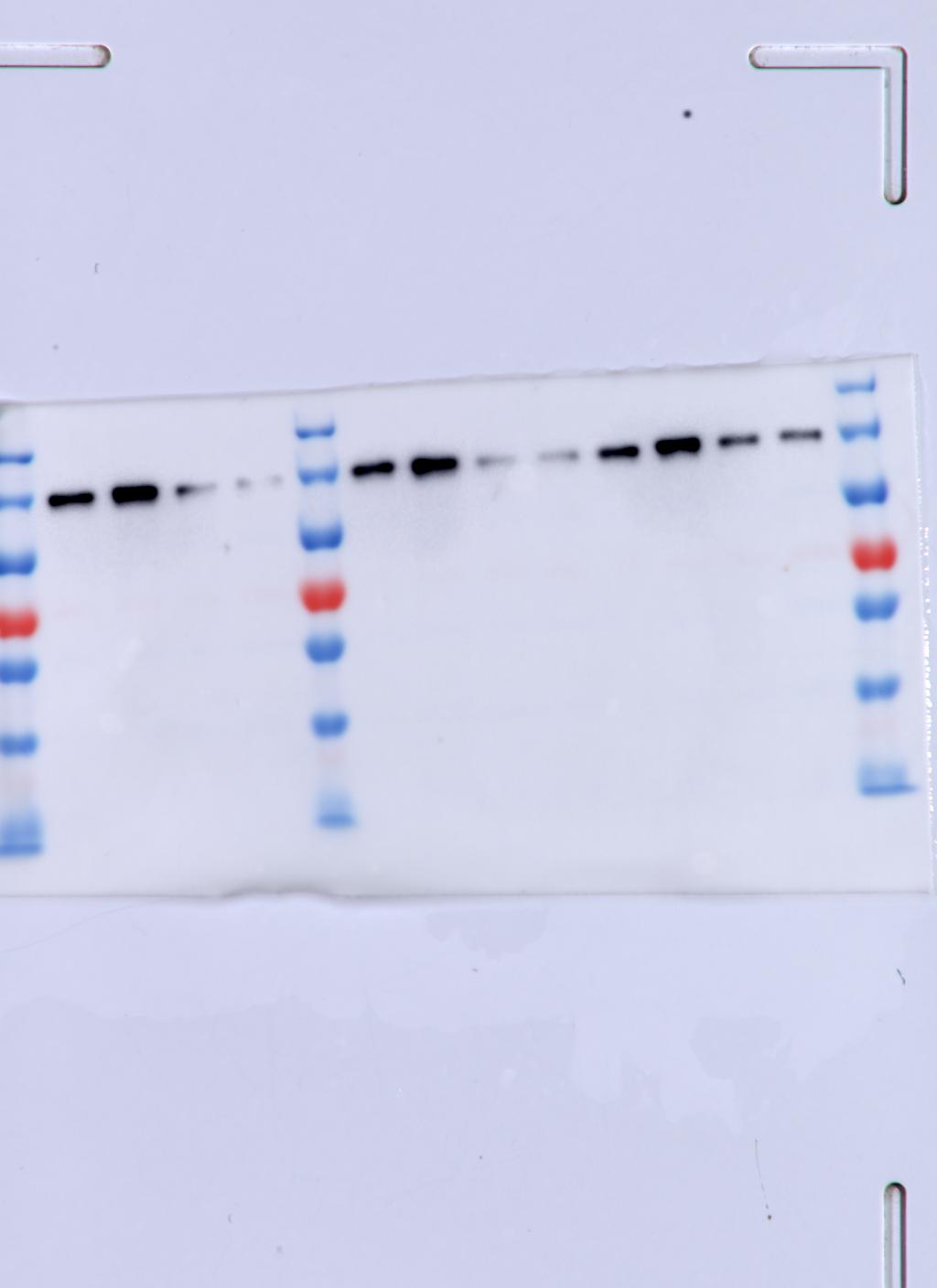

Supplement: Supplementary file 1 [file biology-11-01761-s001.zip › File S3/the relevant data of the vitro experiment to explore the effect and mechanism of cyproterone acetate on ovarian granulosa cell pyroptosis/IRE1/IRE.jpg]

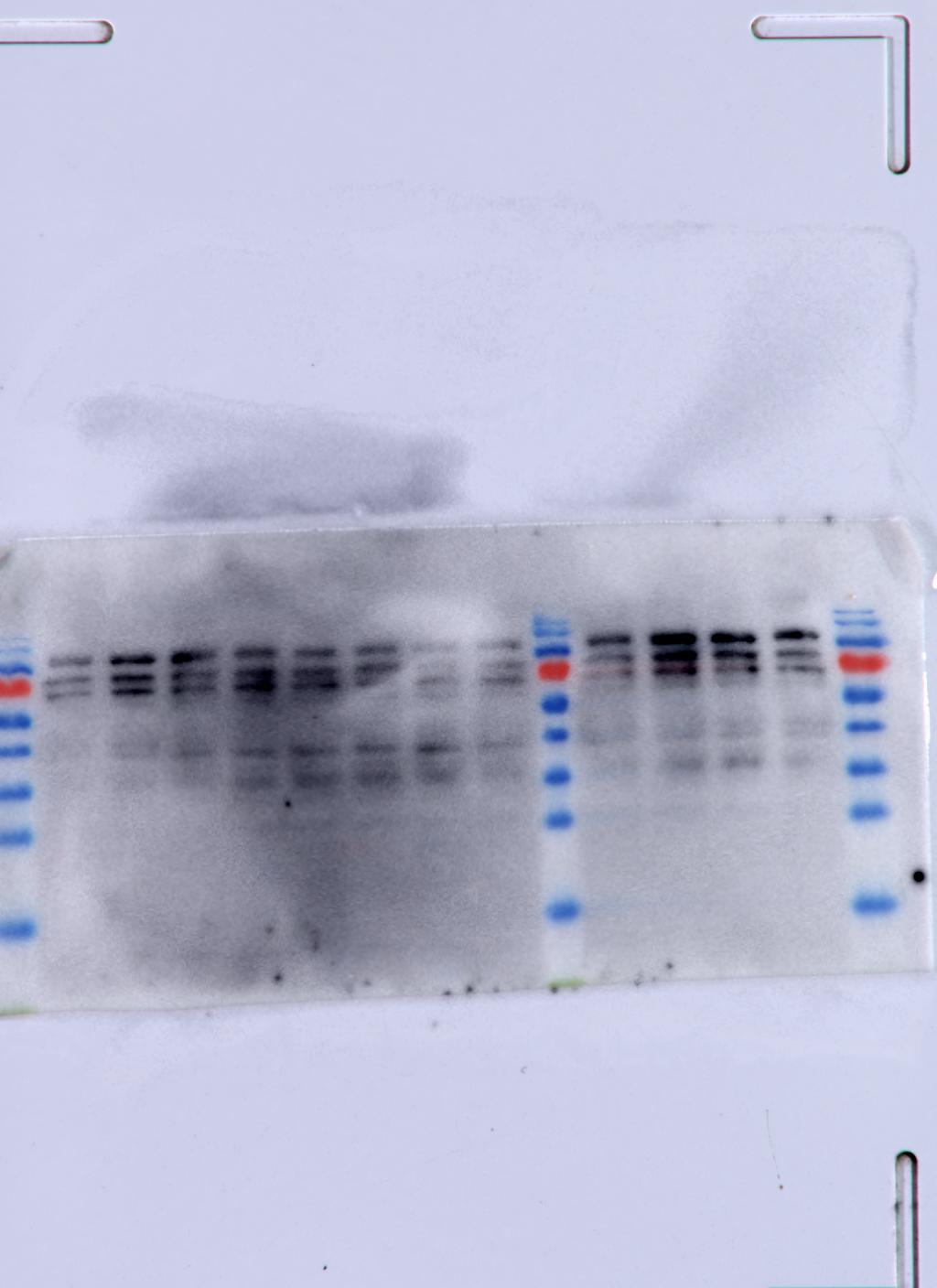

Supplement: Supplementary file 1 [file biology-11-01761-s001.zip › File S3/the relevant data of the vitro experiment to explore the effect and mechanism of cyproterone acetate on ovarian granulosa cell pyroptosis/NLRP3/NLRP3 2.jpg]

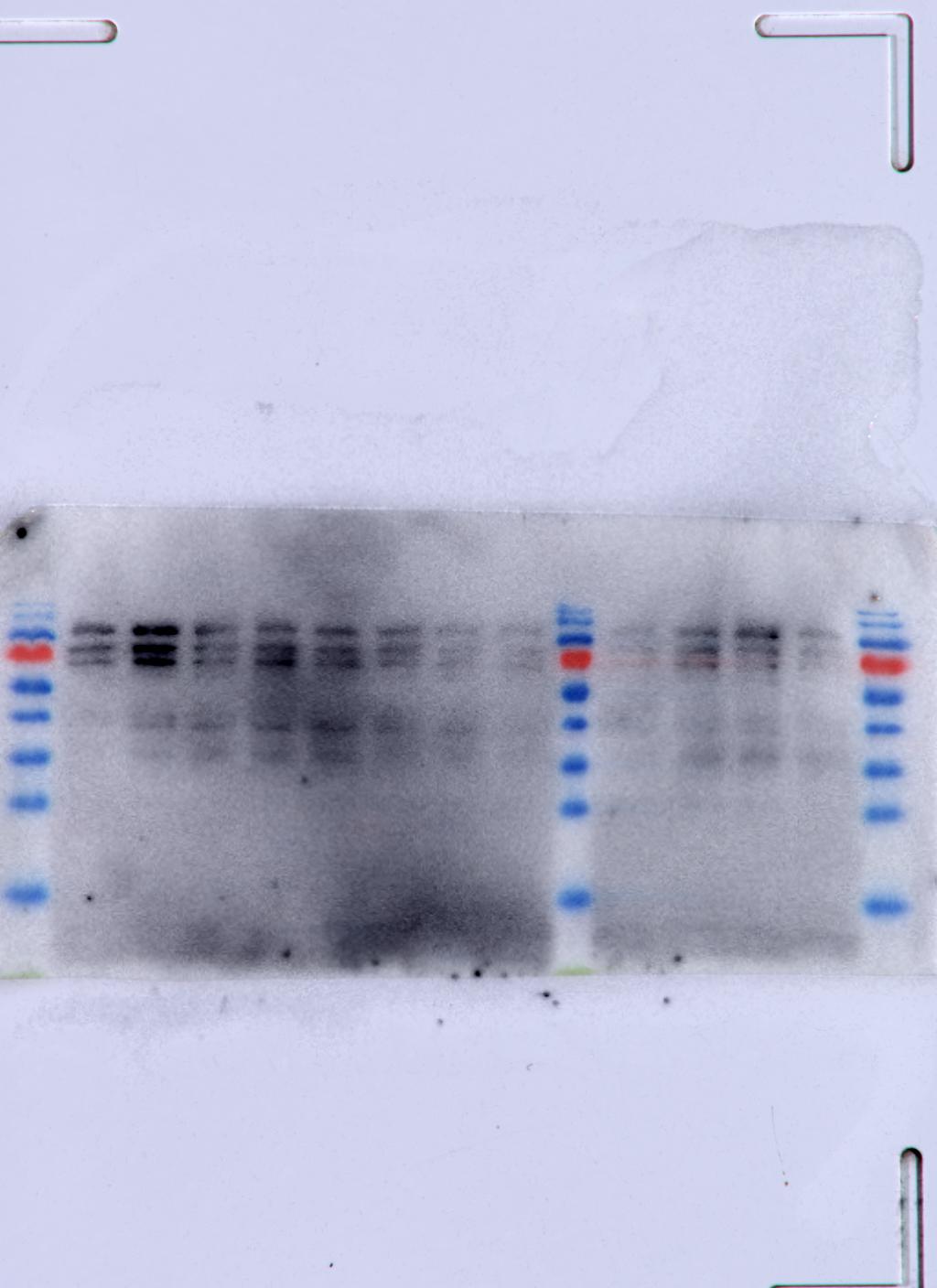

Supplement: Supplementary file 1 [file biology-11-01761-s001.zip › File S3/the relevant data of the vitro experiment to explore the effect and mechanism of cyproterone acetate on ovarian granulosa cell pyroptosis/NLRP3/NLRP31.jpg]

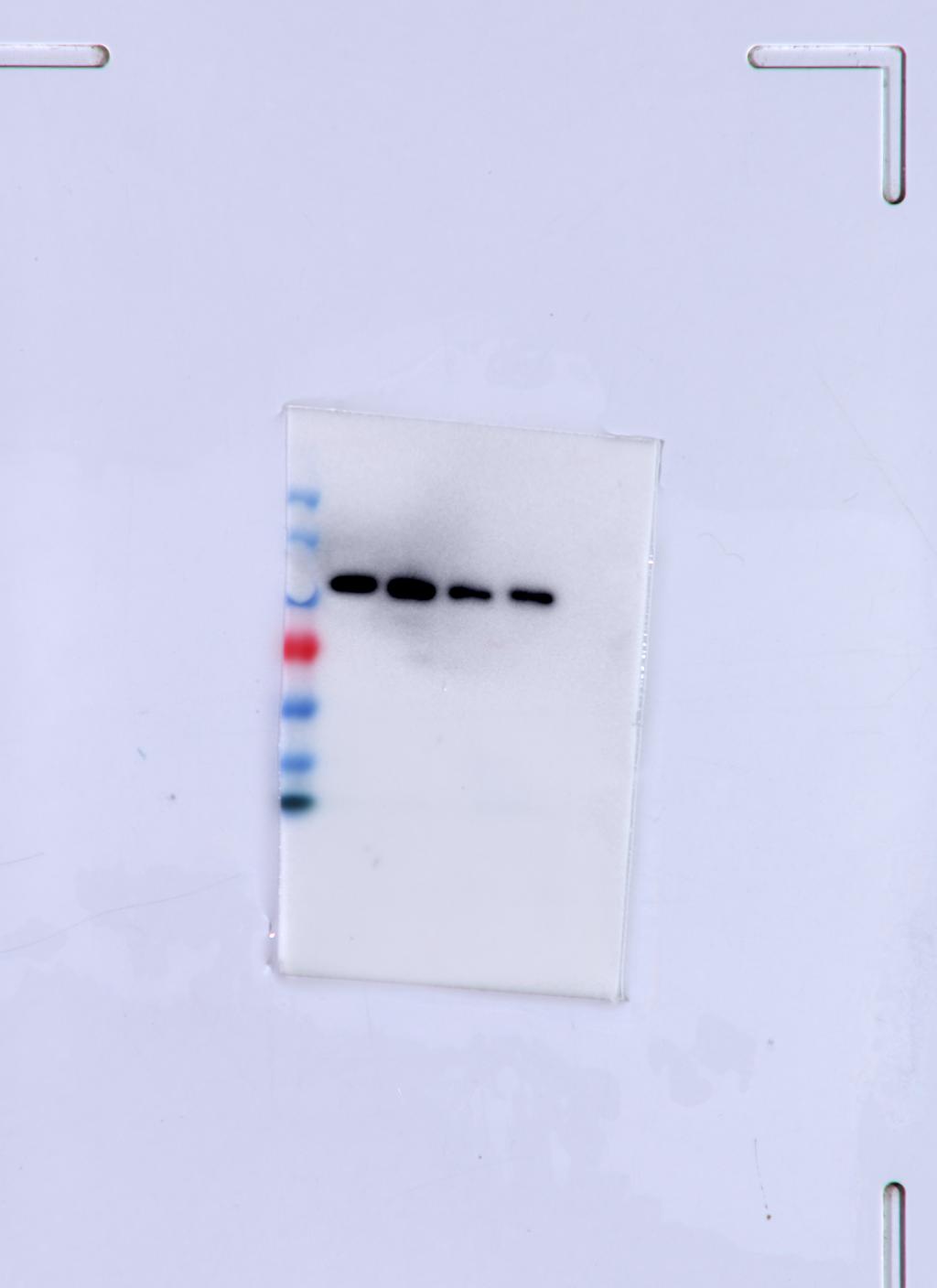

Supplement: Supplementary file 1 [file biology-11-01761-s001.zip › File S3/the relevant data of the vitro experiment to explore the effect and mechanism of cyproterone acetate on ovarian granulosa cell pyroptosis/P-IRE1/P-IRE1.jpg]

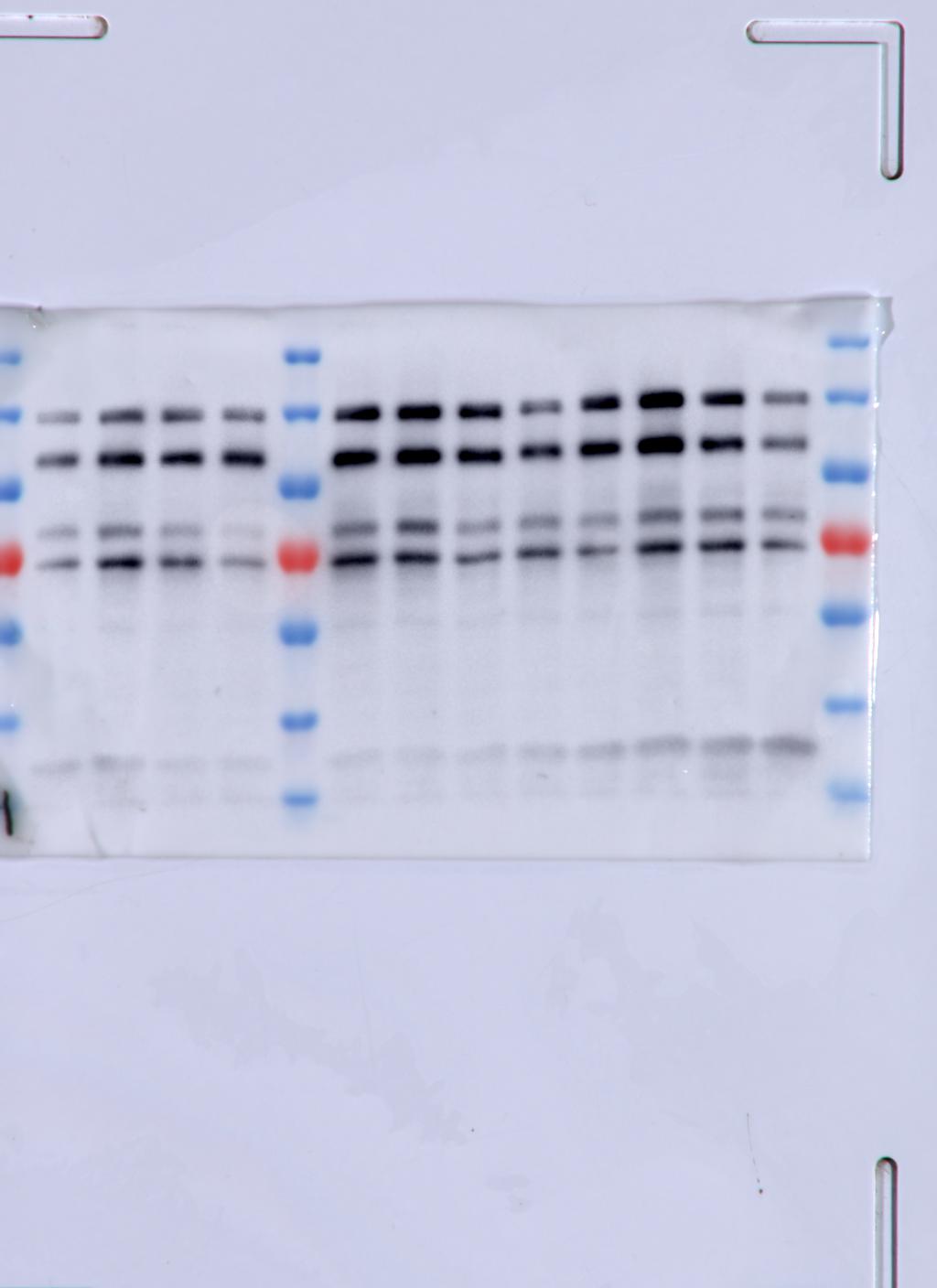

Supplement: Supplementary file 1 [file biology-11-01761-s001.zip › File S3/the relevant data of the vitro experiment to explore the effect and mechanism of cyproterone acetate on ovarian granulosa cell pyroptosis/P-IRE1/pire1 .jpg]

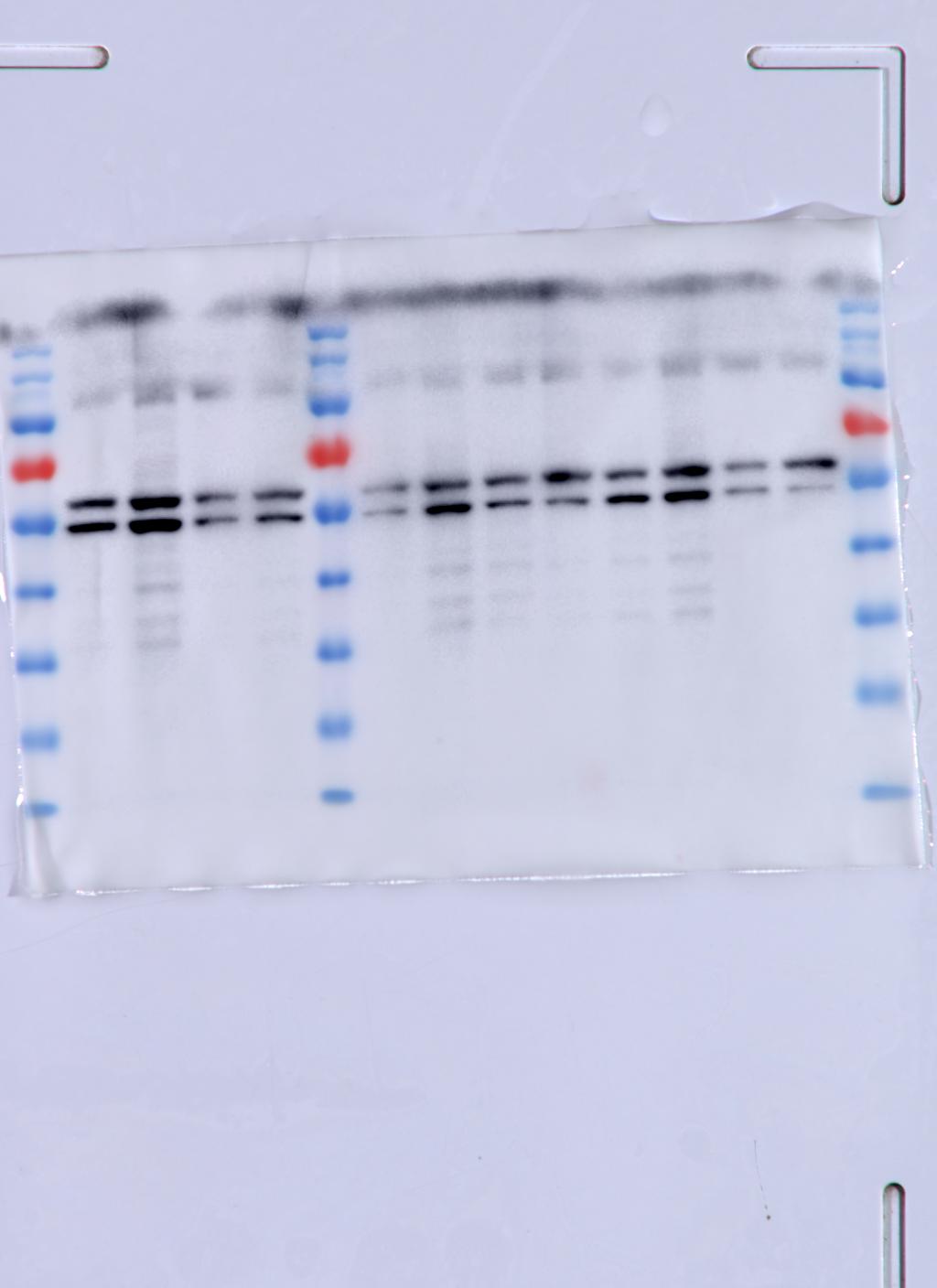

Supplement: Supplementary file 1 [file biology-11-01761-s001.zip › File S3/the relevant data of the vitro experiment to explore the effect and mechanism of cyproterone acetate on ovarian granulosa cell pyroptosis/TXNIP/txnip.jpg]
